# Characterization of markers, functional properties, and microbiome composition in human gut-derived bacterial extracellular vesicles

**DOI:** 10.1080/19490976.2023.2288200

**Published:** 2023-12-01

**Authors:** Chih-Chi Li, Wei-Fan Hsu, Po-Chieh Chiang, Ming-Che Kuo, Andrew M. Wo, Yufeng Jane Tseng

**Affiliations:** a Graduate Institute of Biomedical Electronics and Bioinformatics, College of Electrical Engineering and Computer Science, National Taiwan University, Taipei, Taiwan; b Institute of Applied Mechanics, National Taiwan University, Taipei, Taiwan; c Department of R&D, Reliance Biosciences Inc, New Taipei City, Taiwan; d Department of Medicine, National Taiwan University Cancer Center, Taipei, Taiwan; e Department of Neurology, National Taiwan University Hospital, Taipei, Taiwan; f Department of Computer Science and Information Engineering, College of Electrical Engineering and Computer Science, National Taiwan University, Taipei, Taiwan; g Master’s Program in Smart Medicine and Health Informatics, National Taiwan University, Taipei, Taiwan

**Keywords:** Bacterial extracellular vesicle, extracellular vesicle, stool, fecal, separation, characterization, microbiome, 16S rRNA, metagenomics, personalized medicine

## Abstract

Past studies have confirmed the etiologies of bacterial extracellular vesicles (BEVs) in various diseases, including inflammatory bowel disease (IBD) and colorectal cancer (CRC). This study aimed to investigate the characteristics of stool-derived bacterial extracellular vesicles (stBEVs) and discuss their association with stool bacteria. First, three culture models – gram-positive (G+)*Bc*BEVs (from *B.coagulans*), gram-negative (G-)*Ec*BEVs (from *E.coli*), and eukaryotic cell-derived EVs (EEV, from Colo205 cell line) – were used to benchmark various fractions of stEVs separated from optimized density gradient approach (DG). As such, WB, TEM, NTA, and functional assays, were utilized to analyze properties and distribution of EVs in cultured and stool samples. Stool samples from healthy individuals were interrogated using the approaches developed. Results demonstrated successful separation of most stBEVs (within DG fractions 8&9) from stEEVs (within DG fractions 5&6). Data also suggest the presence of stBEV DNA within vesicles after extraction of BEV DNA and DNase treatment. Metagenomic analysis from full-length (FL) region sequencing results confirmed significant differences between stool bacteria and stBEVs. Significantly, F8&9 and the pooled sample (F5-F9) exhibited a similar microbial composition, indicating that F8&9 were enriched in most stBEV species, primarily dominated by *Firmicutes* (89.6%). However, F5&6 and F7 still held low-density BEVs with a significantly higher proportion of *Proteobacteria* (20.5% and 40.7%, respectively) and *Bacteroidetes* (24% and 13.7%, respectively), considerably exceeding the proportions in stool and F8&9. Importantly, among five healthy individuals, significant variations were observed in the gut microbiota composition of their respective stBEVs, indicating the potential of stBEVs as a target for personalized medicine and research.

## Introduction

The gut microbiome, consisting of diverse and complex microorganisms, plays a role in various host interactions and forms of dysbiosis. This can result in various disorders, such as Crohn’s disease, ulcerative colitis, type II diabetes, cardiovascular diseases,^1^ and even neuropsychiatric disorders,^2^ which are often linked by the activation of the immune system and inflammatory responses.^3^ Despite the crucial role of gut bacteria in regulating the gut microenvironment and host health, the underlying mechanisms of this regulation remain largely unknown.^4,5^ Recent studies suggest that bacterial extracellular vesicles (BEVs) secreted by gut commensal, probiotic, and pathogenic bacteria may play a regulatory role, and even have the ability to cross biological barriers into the bloodstream or central nervous system (CNS) than whole-cell bacteria.^6–9^

Extensive research has already been conducted on human EVs, categorizing them into three main groups: exosomes, microvesicles, and apoptotic bodies, based on their size, biogenesis, and cellular release mechanisms. Microvesicles and apoptotic bodies typically fall within the size range of 100 to 1000 nm and 1–4 μm, respectively. In contrast, exosomes exhibit a diameter of 30–150 nm. The challenge arises from the size and density overlap between exosomes and microvesicles (100–150 nm and 1.08–1.19 g/ml) which makes their differentiation intricate. Exosomes are often identified by their content of endosome-associated proteins such as tetraspanins CD9, CD63, and CD81. However, further investigations are needed to elucidate the distinct characteristics of EEVs present in stool.^10,11^

Both pathogenic and commensal bacteria release roughly 20–400 nm BEVs, consisting of membrane-encased particles that spread some of a parent bacterium’s biological material outside the cell. These vesicles contain biological material from bacteria, such as proteins, enzymes, toxins, polysaccharides, DNA, RNA, and peptidoglycan.^12,13^ Bacteria are classified into two types based on the properties of their cell membranes: gram-negative (G-) and gram-positive (G+). G- bacteria can form vesicles in two ways: by blebbing the outer membrane of the bacterium, creating outer-membrane vesicles (OMVs), and through cell explosion, forming outer-inner membrane vesicles (OIMVs) and explosive outer-membrane vesicles (EOMVs).^14^ G+ bacteria create cytoplasmic membrane vesicles (CMVs) through bubbling cell death caused by endolysin.^12,15^

Numerous studies have shown that commensal microorganisms and their metabolites play a role in the maturation of both the intestinal and systemic immune systems.^16,17^ Toll-like receptors (TLR) are a family of innate immune system receptors that help protect against infections^18^ by recognizing the highly conserved motifs in microbes such as pathogen-associated molecular patterns (PAMPs). Some receptors in the TLR family (TLR1, 2, 4, 5, 6, and 10) help detect certain bacterial components and trigger an immune response.^19^ G- BEVs have an inner layer of phospholipids and an outer layer composed of lipopolysaccharide (LPS), which is known to activate TLR4.^14,20^ This type of BEV is rich in outer membrane proteins, such as OmpA and contains periplasmic components. On the opposite, G+ BEVs, also referred to as CMVs, contain both membrane and cytoplasmic components and present lipoteichoic acid (LTA) on their surface, which can activate TLR2.^15,21^ Peripheral blood mononuclear cells (PBMCs), consisting of lymphocytes, monocytes, and macrophages, express most of the TLRs mentioned above. They can be stimulated by LPS, LTA, or other bacterial components to produce proinflammatory cytokines. As a result, they are suitable for studying the presence and functional properties of BEVs.^14,22^

Furthermore, sequencing of DNA transported by stBEVs may disclose its origin and improve our understanding of the gut microbial community. Amplicon sequencing of the 16S ribosomal RNA (rRNA) gene is an accurate and effective method for identifying bacterial taxonomy through sequence-based analysis.^23,24^ Conventional parallel-type short-read sequencers (V3-V4 region) cannot generate full-length V1-V9 reads (FL regions) of the 16S rRNA gene.^25^ Therefore, partial sequences of particular 16S rRNA gene regions are targeted for sequencing, which can result in ambiguous taxonomic classification.^26^ However, when conducting shotgun metagenomic analysis, it is important to consider the availability of adequate amounts of DNA for testing. This can be a challenging requirement when purifying DNA from EV samples.

Recently, stBEVs have been widely acknowledged in the literature for their ability to induce microenvironmental changes, communicate with bacteria or host cells, and even trigger immune responses leading to the development of diseases such as IBD or CRC. However, most of studies on BEVs were conducted using BEVs isolated from in vitro cultures. It remains to be investigated whether the physiological characteristics of BEVs secreted in vitro are comparable to those secreted in vivo. In addition, some studies investigated diseases using stBEVs did not effectively separate EEVs from BEVs, making it challenging to determine the specific functional roles of stBEVs.^4^

To comprehensively understand the role of stBEVs in the human gut, this study utilized a density-based purification method to isolate stool EVs, followed by an interrogation of the characteristics of the purified EVs. Additionally, an optimized procedure for 16S rRNA sequencing of stBEVs’ DNA was developed to analyze the relationship between stBEVs and the stool microbiota. This refined methodology has the potential to facilitate further exploration of the role of stBEVs in various diseases.

## Results

### Correlate stool EVs (stEVs) with EVs from three culture models – NTA, TEM & protein quantification results

**Figure 1.**
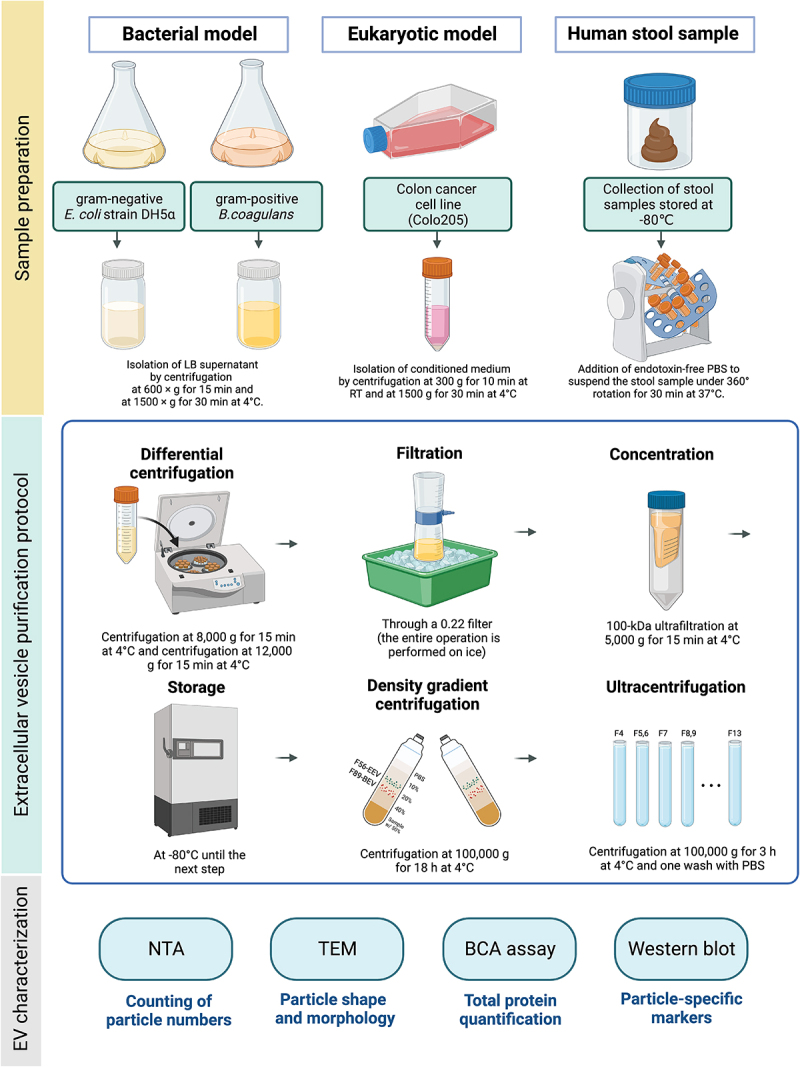
Schematic illustration of extracellular vesicle purification from four different resources.

The stEVs were purified using the same method as the three types of EVs derived from cultured models: G+ BEVs from *B.coagulans*, G- BEVs from *E.coli*, and eukaryotic cell-derived EVs from Colo205 cell (Figure 1). They were referred to as (G+)*Bc*BEVs, (G-)*Ec*BEVs, and EEVs, respectively. The crude extracts of these four sources were separated into 13 fractions using DG and purified into EV fractions through ultracentrifugation (UC) for subsequent experiments (Figure S1).

EVs were isolated from G- bacteria,G+ bacteria, eukaryotic cell culture medium, and human stool specimens by centrifugation, 0.22-μm filter filtration, 100 kDafilter ultracentrifugation, density gradient separation, and ultracentrifugation. After the application of the serial purification strategy, several technologies were used to characterize the EV conditions.

**Figure 2.**
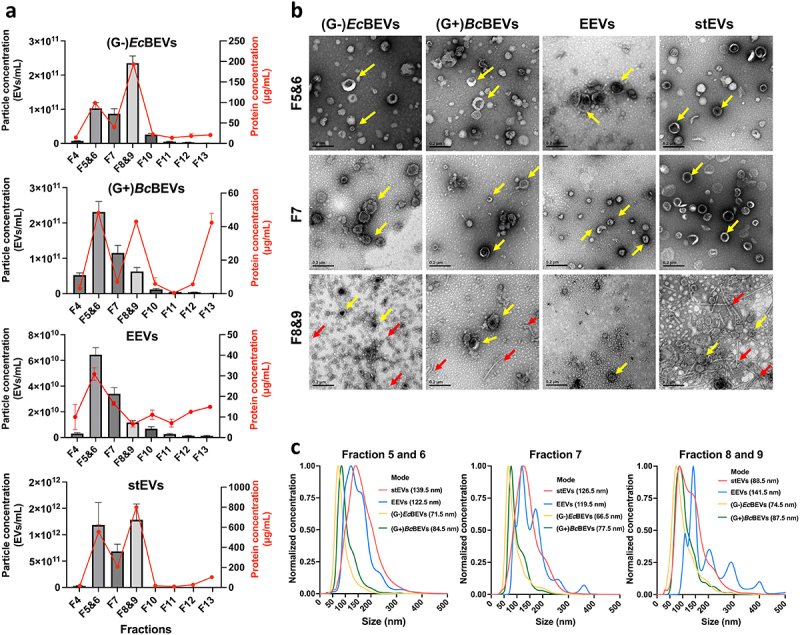
Characterization of EVs derived from four sources (G+ and G- bacterial medium, eukaryotic cell medium, and stool) by the same serial purification method (DG+UC). Samples were divided into several fractions by DG; fractions (F4-F13) were chosen for the characterization of EVs. F5 and F6, F8 and F9 were pooled, respectively. (a) Fractional distribution of particle concentration (left Y-axis, shown in a bar plot) and protein concentrations (right Y-axis, shown with a red line) from (G-)*Ec*BEVs, (G+)*Bc*BEVs, EEVs and stEVs. Results are shown as the means ± SDs of experiments performed in triplicate. (b) Representative TEM images of three major fractions (F5&6, F7 and F8&9) from four resources. (Scale bar = 0.2 µm) The yellow arrows indicate particles with a shape that conforms to the criteriaof EVs, and the red arrows indicate some linear structures, which may be fragments of bacterial flagella or pili. (c) Comparison of the particle sizedistribution, which has been normalized, (by converting the mode of each group to 1) of three main fractions from four different sources determined by NTA. Each line represents the average of two samples with three technical replicates.

Based on the nanoparticle tracking analysis (NTA) results (Figure 2a, left y-axis), the highest particle concentration of (G-)*Ec*BEVs was enriched in DG fractions 8 & 9 (F8&9), while (G+)*Bc*BEVs and EEVs were both in DG fractions 5 & 6 (F5&6). The stEVs showed high particle concentrations in both F5&6 and F8&9 fractions, indicating that EEVs, G+ BEVs, and G- BEVs are predominantly present in these two fractions. Accordingly, it is EEVs and G+ BEVs may be likely to distribute in F5&6, while G- BEVs may be present in F8&9. Furthermore, protein quantification results (Figure 2a, right y-axis) show the concentration of proteins generally resembles closely with the concentration of particles. The high concentration in F13 was likely due to its proximity to the sample source (i.e., the sample was bottom-loading in DG) which may have resulted in residual samples that were not completely separated. Interestingly, although the particle concentration was low in F8&9 for (G+)*Bc*BEVs, the protein concentration was relatively high. Upon further observation of the (G-)*Ec*BEVs and stEVs groups, it was found that the protein concentration was higher in F8&9, while the EEVs group did not show this trend. The above data suggests that samples originating from bacterial sources exhibit higher protein concentrations in F8&9, which may not necessarily correlate with their particle concentrations. This suggests that other bacterial proteins might have been co-purified in this fraction, as confirmed later by TEM and WB data.

Transmission electron microscope (TEM) measurements of three fractions with high particle concentrations (F5&6, F7, and F8&9) revealed that EVs, regardless of their sources or fractions, exhibit size heterogeneity, ranging between 50 and 150 nm (Figure 2b). Additionally, they displayed a distinct cup-shaped morphology. Notably, non-EV structures were observed in F8&9 of (G-)*Ec*BEVs and (G+)*Bc*BEVs and stEV groups, which were also mentioned in the Tulkens et al.^14^ as fragments of pili or flagella produced by bacteria. Based on the size distribution results from NTA, the mode sizes for (G-)*Ec*BEVs and (G+)*Bc*BEVs were small (65–99 nm) across all three fractions, while the mode sizes for EEVs were generally larger (120–150 nm) in all three fractions (Figure 2c). Interestingly, the stEVs showed a mode particle size of 139.5 nm in F5&6, 126.5 nm in F7, and 88.5 nm in F8&9. Notably, in Figure 2c were normalized by assigning the numerical value 1 to the mode of particle size, which does not reflect actual quantities. As a result, the EEVs group in F8&9 exhibited a noise-like peak in the larger particle size range, which was due to the amplification of noise caused by a low particle count, rather than representing the true particle sizes.

Collectively, the NTA data suggest that the particle sizes of stEVs in F5&6 are similar to those of EEVs, while the particle sizes of stEVs in F8&9 are closer to those of (G-)*Ec*BEVs and (G+)*Bc*BEVs groups. This indirectly suggests a higher presence of EEVs in stEVs from F5&6 and a higher presence of G- BEVs in F8&9. However, further discussion and characterization of EV features are required.

### Correlate stEVs with EVs from three culture models – WB

**Figure 3.**
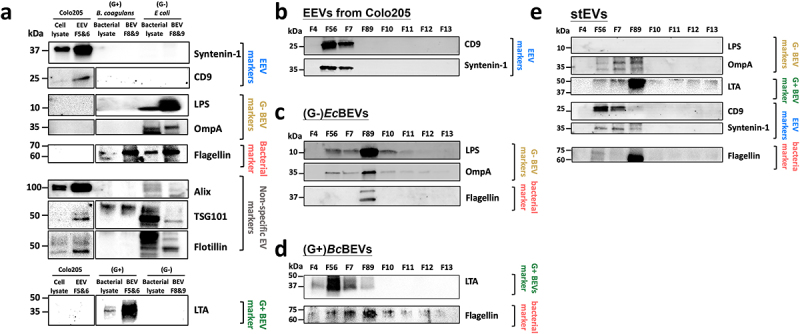
Western blot of the separated fractions by DG show enrichment of G- BEVs, G+ BEVs, and EEVs. (a) Investigating marker specificity by analyzing cell or bacterial lysates and their EVs across three cultured models. Representative western blot analyses of (b) EEVs, (c) (G-)*Ec*BEVs, (d)(G+)*Bc*BEVs, and (e) stEVs specimens fractionated by DG+UC separation. Ten micrograms of F5&6, F7 and F8&9 were loaded in each lane, and 30 µl of the other fractions was loaded. The blot was probed with antibodies against the EEV markers CD9 and syntenin-1; the G- BEV markers LPS and OmpA; the G+BEV marker LTA; and the bacterial marker flagellin, The non-specific EV markers flotillin, TSG101, and Alix.

Using multiple markers, Figure 3a shows WB results for cell or bacteria lysates from three different models and their corresponding EVs. Syntenin-1 and CD9 signals are only found in the Colo205 group, while LPS and OmpA signals are specific to the (G-)*E.coli* group. LTA signals are unique to the (G+)*B.coagulans* group. Flagellin signals appear in both (G-)*E.coli* and (G+)*B.coagulans* groups. Surprisingly, signals for Alix, TSG101, and Flotillin were detected not only in the Colo205 group but also in the other two bacterial groups. This indicated that these three markers might exhibit nonspecific binding to bacterial proteins. Consequently, we designated these markers as nonspecific EV markers and omitted them from the ensuing discussion regarding EEVs and BEVs.

For EEVs (Figure 3b), EEV markers like CD9, Syntenin-1 exhibit strong signals in F5&6. These signals align well with the NTA results in Figure 2a, confirming the successful isolation of EEVs in F5&6 through this purification method. In the case of (G-)*Ec*BEVs (Figure 3c), results reveal strong staining for G- BEV markers (LPS and OmpA) and bacterial flagellar fragments (Flagellin) in F8&9. This suggests that the purification method effectively isolates (G-)*Ec*BEVs in F8&9 but may still contain some flagellar contamination. For (G+)*Bc*BEVs (Figure 3d), G+ BEV marker (LTA) shows a strong positive stain in F5&6, which correlates with the corresponding NTA results with a clear signal in F5&6, shown in Figure 2a. Flagellin also mainly appeared in F8&9, which may explain why (G+)*Bc*BEVs exhibited high protein concentrations in F8&9 in Figure 2a.

For stEVs (Figure 3d), the G- BEV marker OmpA was detected in F8&9 as expected, while no LPS signal was observed. The LTA marker for G+ BEVs unexpectedly appeared in F8&9 instead of F5&6, which differs from the result of (G+)*Bc*BEVs. The variation might result from differences in particle density between (G+)*Bc*BEVs and G+ stBEVs, suggesting that BEVs from each bacterial species could have unique densities. This density distinction isn’t solely dictated by G+ or G- characteristics. The metagenomic analysis of stBEVs data (Figure 7b) supported this notion. Among the EEV markers, CD9 and Syntenin-1 were detected in F5&6 as expected. Flagellin were detected in F8&9 as expected. In summary, we observed the presence of CD9 and syntenin-1 in F5&6, whereas OmpA and LTA were identified in F8&9. This suggests that stEEVs are concentrated in F5&6, while stBEVs are enriched in F8&9. However, F8&9 still contain a significant amount of flagella, which was removed for further functional assays later.

### Stool BEVs distribution can be assessed by combining endotoxin measurements, immunoassays, and NTA

**Figure 4.**
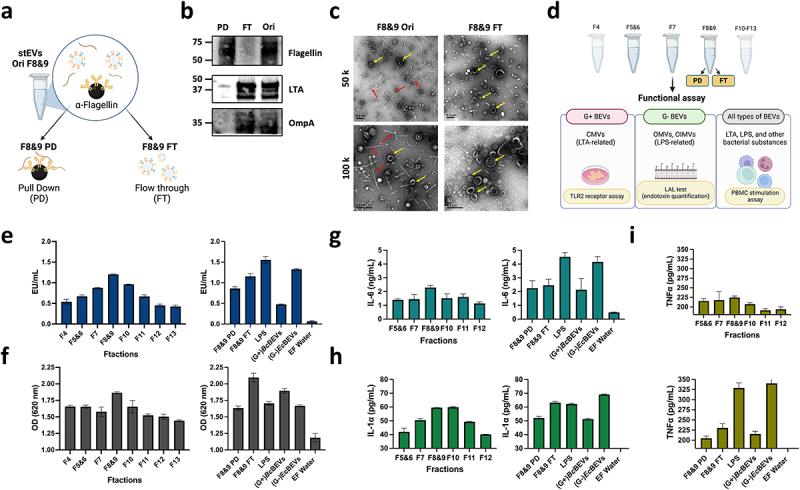
Determining the location and biological traits of stool-derived EVs through functional assays. (a) The image illustrates the process of immunoprecipitation (IP) and the naming origin of samples tested for F8&9 in stEVs, grouped as Pull Down (PD), Flow-through (FT), and untreated F8&9 (Ori). (b)The WB shows the expression levels of flagella and G+BEVs vs. G-BEVs in F8&9 after IP. (c) Representative TEM images from F8&9 Ori and F8&9 FT are displayed. The scale bar is 0.2 µm. Yellow arrows highlight EVs , while red arrows indicate flagella or pilifragments. The upper image is magnified at 50,000x, and the lower image at 100,000x. (d) This diagram explains the evaluation of functional traits in separated stool BEV samples and their associated result figures.EV characteristics were examined from various fractions using tests (e) LALtest, (f) TLR2 receptor assay and (g, h, i) PBMCs stimulation. F8&9 PD (flagella representation) and F8&9 FT (pureF8&9 BEVs), (G+)*Bc*BEVs from F5&6, (G-)*Ec*BEVs from F8&9, LPS were included as a positive control, and endotoxin-free (EF) water as a negative control. (g) IL-6, (h) IL-1α, and (i) TNFα were the cytokines tested in stimulated PBMCs. The figure includes datafrom three technical replicates, with error bars representing standard deviations.

Figure 4a displayed the initial stEVs in F8&9 (F8&9 Ori) subjected to immunoprecipitation (IP) for obtaining flagella pulldown (PD) and flow-through (FT). Subsequently, the elution of proteins from PD was performed. Figure 4b presents the WB results of stEVs F8&9 PD, FT, and Ori. Flagellin is enriched in the PD group, while LTA and OmpA are enriched in the FT group. Furthermore, in Figure 4c, TEM images clearly show that most of flagella have been effectively removed, whether magnified at 50,000× or 100,000×. The combined evidence from Figures 4(b,c) demonstrates that this IP method successfully separates Flagella fragments from BEVs. Consequently, in subsequent experiments, F8&9 PD was recognized as the fraction containing flagella, and F8&9 as the purified stEVs.

Figure 4d illustrates the design of subsequent functional assays, targeting stEVs from F4-F13. Additionally, separate experiments are conducted for F8&9 PD and FT, which utilize an equivalent protein quantity as F8&9. Moreover, LPS, (G+)*Bc*BEVs from F5&6, and (G-)*Ec*BEVs from F8&9 (after flagellin removal by IP) serve as positive control groups. Similarly, both BEVs groups are loaded with a comparable particle concentration as the stEVs groups, while endotoxin-free (EF) water is used as a negative control. Limulus amebocyte lysate (LAL) assay is a sound method for accurately measuring endotoxin (LPS) in a sample.^27^ We utilized this characteristic to measure the relative abundance of G- BEVs based on the signal intensity from this assay, as G- BEVs carry LPS as a marker. In the stEV group, it was clearly observed that G- BEVs were mainly present in F8&9 (Figure 4e, left). Furthermore, F8&9 PD exhibited a slightly lower signal compared to F8&9, while F8&9 FT showed a signal similar to that of F8&9. It was observed that the (G-)*Ec*BEVs exhibited a higher signal, while the (G+)*Bc*BEVs displayed a comparatively lower signal (Figure 4e, right). It can be inferred that the LAL test produces a high signal in samples containing LPS. Therefore, despite the absence of specific antibody staining for LPS in the WB image of stEVs, this experiment confirms that LPS primarily exists in stEVs from F8&9.

In the results of the TLR2 receptor assay, focusing solely on the stEV groups, it can be observed that F8&9 still exhibit a slightly higher signal (Figure 4f, left). Furthermore, F8&9 FT shows a slightly higher signal compared to F8&9. It is hypothesized that in the case of F8&9 FT, the removal of flagellin resulted in increased purity, leading to a higher quantity of BEVs in samples of the same concentration. Furthermore, at equivalent concentrations, (G+)*Bc*BEVs display a stronger signal than (G-)*Ec*BEVs (Figure 4f, right). This indicates that G+ BEVs in F8&9 FT likely substantially contribute more to the signal than G- BEVs.

Some studies^28^ have suggested that BEVs have the ability to activate human PBMCs in *ex vivo* assays due to their expression of bacterial markers such as LPS, TLA, or flagella. This experimental approach was aimed at measuring the production of the proinflammatory cytokines IL-6, IL-1a, and TNF-α in PBMCs after activation to provide a functional basis for determining the relative abundance of BEVs. When focusing solely on the stEV groups, the three cytokines still exhibit the highest signal in F8&9 (Figure 4g-i, left). In contrast, there isn’t a significant difference observed between F8&9 PD and FT groups when compared to F8&9. Notably, LPS and (G-*)Ec*BEV demonstrate a more pronounced impact on this immune response, resulting in significantly higher signals, whereas (G+)*Bc*BEVs have a comparatively smaller effect (Figure 4g-i, right). Accordingly, it is evident that G- BEVs stimulate PBMCs to a greater extent compared to G+ BEVs and Flagellin.

Based on the cumulative findings, we proposed that F8&9 predominantly capture a significant portion of G+ and G- stBEVs. Therefore, we referred to the stEVs from F8&9 as stBEVs for the subsequent analysis.

### Exploration of purification strategy for stBEVs DNA and investigation of fragment selection targeting 16S rRNA gene regions

**Figure 5.**
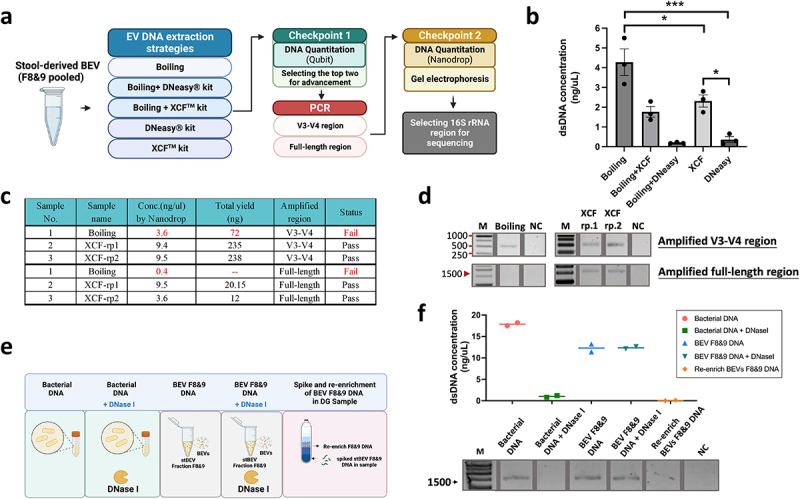
Optimization of stool-derived BEV DNA extraction for 16S gene amplification. (a) Illustration of the BEV DNA extraction, PCR gene amplification, and DNA qualitycontrol strategies. (b) Total DNA yield (ng/µL) obtained from different DNA extraction methods measured by Qubit 2.0, mean ±SD, one-way ANOVA test, *p < 0.05, ***p < 0.0005. (c, d) DNA extracted from stBEVs using two methods, Boiling and XCF, was amplified by PCR targeting the V3-V4 region and the FL region. The total amplified DNA yield (ng) was measured using a NanoDrop system, and electropherograms were used to assess results from the quality control. The XCF underwent two repeated PCR experiments, referred to as rp1 and rp2. (e) This figure shows five tests to determine if stBEV DNA is enclosed in the membrane. It includes pre-processing, DNA extraction using the XCF kit, PCR amplification targeting FL regions, (f) measuring DNA concentration with NanoDrop, and verifying DNA quality through gel electrophoresis.

Figure 5a illustrates the experimental design for this section, which includes EV DNA extraction strategies, PCR amplification of different regions of 16S rRNA, and two checkpoints to determine the most appropriate approach for each step. During the optimization phase of stBEV DNA extraction, a pooled sample of F8&9 was used as a sample. After DNA extraction, using Qubit 2.0 system, it was found that the Boiling method presented the highest DNA yield, followed by the XCF kit method. Therefore, these two methods were selected for further bacterial DNA quality testing experiments (Figure 5b). After amplifying the V3-V4 and FL regions of the 16S rRNA gene, it was found that the Boiling method did not produce enough DNA for further analysis under either gene amplification strategy. In contrast, the XCF kit-extracted samples show sufficient DNA quantities under both gene amplification strategies. This indicates that the absence of DNA purification in the Boiling method led to significant contamination, or alternatively, DNA fragmentation that adversely affected gene amplification efficiency (Figure 5c). Based on the electrophoresis results, the Boiling group showed PCR bands in V3-V4, but the amount of DNA did not reach the required threshold. In contrast, no bands were observed in the FL group, and this group was excluded from further analyses. On the other hand, the XCF kit group exhibited clear bands under both gene amplification strategies, indicating successful gene amplification (Figure 5d). As a result, the XCF kit method was chosen for DNA purification in subsequent stBEV studies. Since successful amplification was achieved for both the V3-V4 and FL regions of the 16S rRNA, both groups proceeded to the next sequencing experiment for further comparison.

In addition, the origin of BEV DNA, whether it originates from within or outside the membrane, was investigated. DNA purified from E. coli lysate (bacterial DNA) was used as a sample. Equal amounts of DNase I were separately added to both bacterial and BEV samples. Furthermore, we explored whether soluble DNA could be enriched in the DG’s F8&9. Therefore, an additional experimental group was included to test this phenomenon (Figure 5e). DNA quantification and electrophoresis results showed that equal DNase treatment led to the lysis of Bacterial DNA, preventing PCR amplification. In contrast, it had no impact on BEV F8&9 DNA. Additionally, no signal was observed for re-enriched BEVs F8&9 DNA (Figure 5f). These findings strongly suggest that the amplified BEV DNA exclusively originates from the intramembrane of BEVs.

### The microbiota composition analysis of stBEVs was investigated using two different 16S rRNA sequencing techniques

**Figure 6.**
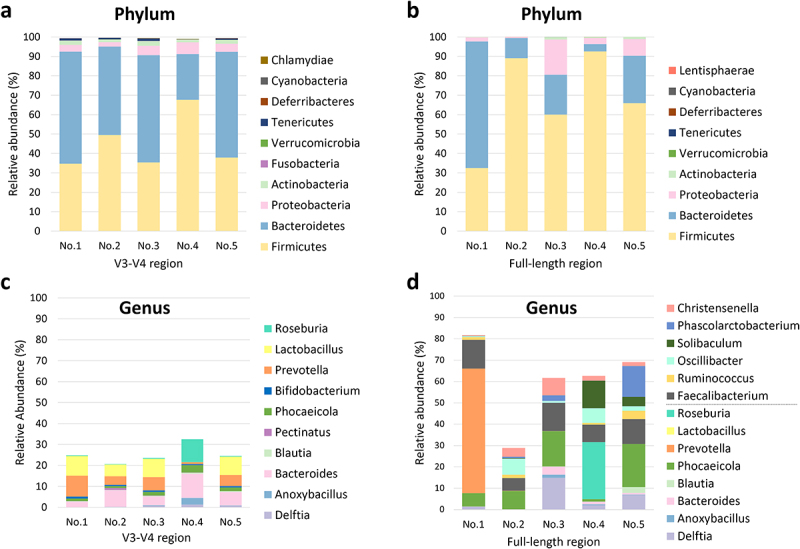
Comparison of taxonomic assignments from stBevs at the phylum and genus levels using the 16S rRNA V3-V4 region, analyzed with the Illumina platform, and the 16S rRNA FL region, analyzed with the PacBio platform. The figures present comparisons among the top ten phylum-level (a, b) taxonomic assignments in terms of relative abundance according to the sequencing results for the V3-V4 region and FL region. In the genus-level plots (c, d), the category ”Other” was excluded. Additionally, for the FL region, in addition to showing the top ten genera, the genera for which the V3-V4 region was sequenced were also included in the bar graph for comparison. No.1 to No.5 represent stBEVs derived from five healthy individuals.

To ensure optimal microbial composition analysis in stBEVs samples, we utilized DNA extracted from stBEVs purified from F8&9 fractions obtained from five healthy individuals as the source material. Five stBEV samples were each divided for sequencing, targeting the V3-V4 region and the FL region. Figure 6(a,b) demonstrate that both sequencing methods primarily identified *Firmicutes*, *Bacteroidetes*, *Proteobacteria*, and *Actinobacteria* as the dominant phyla. These phyla accounted for approximately 98% to 100% of the microbial composition in both groups. Moreover, *Firmicutes* and *Bacteroidetes* were found to be the dominant components, accounting for 90–93% in the V3-V4 region group and 80–97% in the FL region group. On the other hand, it was observed that within the phylum *Firmicutes*, the FL region group exhibited a higher average relative abundance (68 ± 24%) compared to the V3-V4 region group (45 ± 13.9%), with greater variability among the samples. These results suggest that the FL region sequencing method more clearly reveals differences among the samples at phylum level.

At the genus level, we excluded the unclassified group labeled as “Others” and focused solely on the genera that could be classified in both groups (Figure 6(c,d)). The relative abundance of the genera detected by sequencing the V3-V4 region accounted for 20–30% of the total abundance. In comparison, that of genera detected by sequencing the FL region accounted for an average of 60%, with greater diversity of genera detected. Among the highly abundant genera, shared by bothㄜ groups, are *Delftia*, *Anoxybacillus, Bacteroides, Blautia*, *Phocaeicola*, *Lactobacillus*, and *Roseburia*, although not expressed in every sample. In the V3-V4 region group, *Bacteroides* (average 6.57 ± 3.48%) and *Prevotella* (average 5.31 ± 3.33%) exhibited relatively higher expression levels across all five samples.

Only *Faecalibacterium* exhibited relatively high expression levels in the FL region group across all five samples (average 10.5 ± 3.28%). On the other hand, other genera showed variations in expression levels among individuals. These results indicate that sequencing targeting the FL region provides higher overall richness in the detected genera compared to sequencing targeting the V3-V4 region. Consequently, in subsequent experiments, this sequencing method was used to investigate further the interrelationship between the microbiota of stEVs from different fractions and the gut microbiota.

### Differential gut microbiome composition in stool samples and stBEVs from various fractions and their interrelationship

Individual samples were collected from five healthy individuals, and 16S rRNA sequencing targeting the FL region was performed to analyze gut microbiota composition.

**Figure 7.**
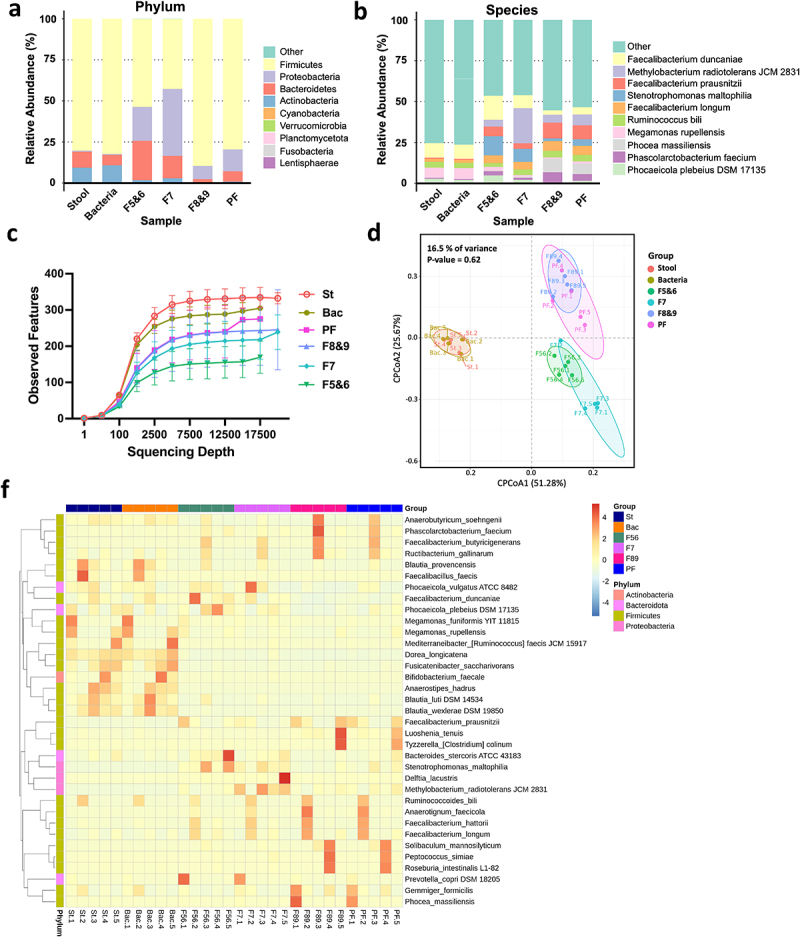
The microbiome in stool and various stBEV fractions from five healthy individuals was analyzed, including assessments of α-diversity and β-diversity. This comparison displays the relative abundance (% of total 16S rDNA gene sequences) of gut microbes at the phylum level (a) and species level (b) in stool, stool bacterial lysate (Bacteria), and stBEV fractions: F5&6, F7, F8&9, and pooled-fractions F5-F9 (PF). The data represents the average results from five healthy individuals, presented in a grouped format. (c) Rarefaction curves compare thetotal diversity among the six groups. Stool (St), Bacteria (Bac), and stBEV groups (PF, F8&9, F7,F5&6). (d) CPCoA plot illustrates bacterial β-diversity, the percentage indicatesthe contribution of group information to sample variations, ranging from 0% to100%. The p-value assesses whether group information influences the explanation of some sample differences (f) Heatmap shows the 35 bacterial species with thegreatest differences in relative abundance between stool, stool bacteria and stBEV groups at the species level.

Figure 7(a,b) represent the averaged results of all five samples, while individual sample results for phylum and species are presented in Figure S3. Figure 7(a,b) show the top 10 most prevalent taxa within the stool, stool bacteria (bacteria), and stBEVs groups (F5&6, F7, F8&9, PF), categorized by phylum and species levels.

At the phylum level, within the *Firmicutes* category, the proportions are as follows: stool, bacteria, F5&6, F7, F8&9, and PF account for 80.2%, 82.3%, 53.5%, 42.6%, 89.6%, and 79.5%, respectively. Notably, *Firmicutes* exhibited high abundance in stool, bacteria, F8&9, and PF groups. In the *Bacteroidetes* category, the respective proportions, in the same order as mentioned above, are: 9.8%, 6.4%, 24%, 13.7%, 2.2%, 6.3%. Notably, F5&6 exhibit a higher proportion of *Bacteroidetes* compared to other groups, with F7 ranking second. In the *Proteobacteria* category, the respective proportions, in the same order as mentioned above, are: 0.7%, 0.5%, 20.5%, 40.7%, 8%, 13.3%. Notably, there is an interesting observation with F7 exhibiting a significantly higher proportion of *Proteobacteria* compared to other groups, with F5&6 ranking second. In the *Actinobacteria* category, the respective proportions are as follows: 9.3%, 10.7%, 1.7%, 2.9%, 0.2%, 0.8%, in the order mentioned. The highest proportion of *Actinobacteria* is found in the Stool and bacteria groups, suggesting that Actinobacteria may not produce EVs as readily.

At the species level, we observe similarities in composition: stool and bacteria, F5&6 and F7, and F8&9 and PF, when grouped pairwise. Six groups consistently demonstrated a substantial presence of *Faecalibacterium duncaniae* (8.9%, 8.7%, 14.7%, 8.0%, 2.7%, 4.4%), indicating both their abundance and capacity for BEV production. Moreover, it was observed that the majority of these BEVs were predominantly found in F5&6, characterized by lower density. *Megamonas rupellensis* is only predominantly present in Stool and Bacteria (6.2%, 6.8%), indicating a high abundance in the gut microbiota but infrequent BEV production. *Faecalibacterium prausnitzii* exclusively occupies F8&9 (9.7%), and despite their lower abundance in the gut microbiota, these strains tend to produce higher-density BEVs. *Faecalibacterium longum* is predominantly found in the distribution of stBEVs across all groups (4.6% to 5.9%). It is noteworthy that *Stenotrophomonas maltophilia* shows higher expression in both F5&6 and F7 (11.7% and 8%, respectively), whereas *Phocaeicola plebeius DSM 17,135* exhibits elevated expression exclusively in F5&6 (4.9%). Additionally, *Methylobacterium radiotolerans JCM 2831* was exclusively detected in F7 at a significant level (21.5%). We discuss bacterial strains with high expression in at least two or more samples (Figure S3). Strains with isolated cases of high expression are not included in this discussion. The stool and bacteria group had a high species diversity, resulting in most of its members being classified as “other.”

The rarefaction curves confirmed that the sequencing depth was sufficient to assess the representative microbial composition of the six samples. When using the same sequencing depth for all samples, diversity is highest in the Stool group, followed by the Bacteria group (Bac), and similar levels in the PF and F8&9 groups. The F7 group has slightly lower diversity, while F5&6 exhibits the lowest diversity (Figure 7c). This trend is consistent with the α-diversity indices (Table 1).

**Table 1.** α-diversity indices of stool, bacteria, different stBevs fractions determined by FL 16S rRNA sequencing.

	Observed features	Shannon entropy	Simpson	Pielou evenness	Menhinick	Margalef	Faith pd	Goods coverage
Stool	336.4 ± 27.61	7.11 ± 0.28	0.98	0.85	2.33 ± 0.25	33.73 ± 2.87	11.98 ± 1.05	1
Bacteria	291.0 ± 27.05	6.95 ± 0.25	0.98	0.85	2.31 ± 0.26	29.96 ± 2.66	10.98 ± 1.07	1
PF	247.0 ± 75.43	5.83 ± 0.67	0.96	0.74	1.82 ± 0.49	25.04 ± 7.49	14.59 ± 2.60	1
F8&9	246.2 ± 96.47	5.33 ± 0.73	0.91	0.68	1.72 ± 0.65	24.68 ± 9.64	15.09 ± 3.04	1
F7	221.8 ± 54.44	5.31 ± 0.28	0.94	0.69	1.57 ± 0.38	22.30 ± 5.48	13.48 ± 1.95	1
F5&6	159.6 ± 44.25	4.83 ± 0.21	0.92	0.67	1.16 ± 0.31	16.10 ± 4.44	10.44 ± 0.96	1
	actual species number	score ↑, diversity ↑			community species richness		score ↑, sample authenticity ↑	

We analyzed β-diversity using Constrained Principal Coordinates Analysis (CPCoA), which integrates PCoA and RDA methods while considering group information to identify key differences between groups. We assessed sample similarity and found that group information contributes only 16.5% of the variance in this sample, with a non-significant P-value of 0.62. This suggests that group categorization has minimal impact on our analysis results (Figure 7d). Hence, this plot illustrates similar findings to Figure 7b, showing composition similarities when grouped pairwise: stool and bacteria, F5&6 and F7, F8&9 and PF.

Figure 7f shows the hierarchical clustering and heatmap of the 35 species with the highest relative abundance. Although the stool, F8&9, and PF groups all presented a high proportion of *Firmicutes* at the phylum level, their bacterial and BEV compositions were markedly different. This suggests that not every bacterial species with high gut abundance produces BEVs. Furthermore, while most of the species in PF are contributed by F8&9 (consistent with WB and functional assay results), it is also observed that F5&6, F7, and F8&9 have species present under these three different densities. This suggests that each strain’s BEV may vary, explaining why the signal of LTA in (G+)*Bc*BEV and stEVs is not found in the same fractions as observed in the WB results. Taken together, upon observing each grouping, significant inter-individual variability was evident. However, individuals displayed similar patterns when grouped pairwise, affirming the technique’s robustness, with differences primarily arising from individual variability.

### Differences in metagenomic analysis between various fractions of stBEVs and stool samples were assessed using Welch’s t-test and LEfSe analysis

**Figure 8.**
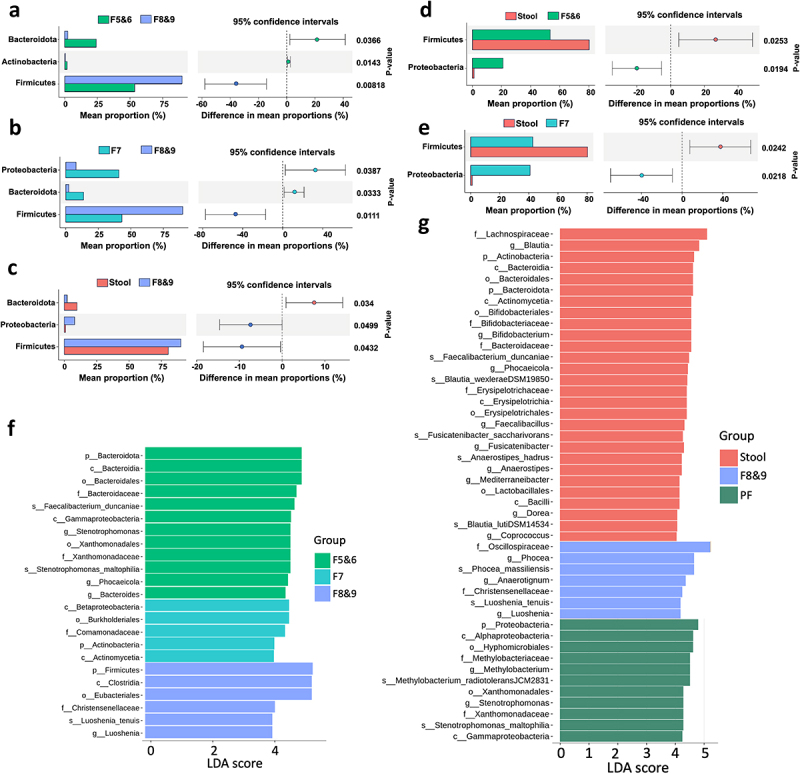
Significant differences in the microbiota of stool DNA and stBEV DNA in different fractions were identified by metagenomic analysis. Investigation of microbial composition differences involved Welch‘s t-test comparisons between (a) F5&6 vs. F8&9, (b) F7 vs. F8&9, (c) Stool vs. F8&9, (d) Stool vs. F5&6, and (e) Stool vs. F7. The left y-axis represents phyla, with mean abundance on the x-axis. Colored dots highlight groups with higher phylum abundance, and error bars depict the 95% confidence intervals for group differences. Significance levels are shown on the right y-axis. LEfSe analysis demonstrated significant bacterial distinctions in fecal microbiota for (f) F5&6 vs. F7 vs. F8&9 and (g) Stool vs. F8&9 vs. PF, with LDA scores (log10) > 4 and P value <0.05 displayed.

Figure 8(a-e) present the results of Welch’s t-test, comparing pairwise group data at the phylum level. F8&9 serves as the primary concentration of BEVs, hence it is compared to F5&6, F7, and stool groups. F5&6 showed significantly higher proportions of *Bacteroidota* and *Actinobacteria* compared to F8&9 (p-values = .037 and .014, respectively). Additionally, F7 exhibited a notably higher abundance of *Proteobacteria* and *Bacteroidota* compared to F8&9 (p-values = .039 and .033, respectively). Conversely, F8&9 displayed a significant composition of *Firmicutes*, with p-values = .008 and .011 in contrast to F5&6 and F7, respectively. Compared to stool, F8&9 exhibited a notably higher abundance of *Bacteroidota* (p-value = .34). Additionally, both F5&6 and F7 showed significantly higher levels of *Proteobacteria* compared to stool (p-values = .019 and .02, respectively). In summary, when comparing stBEV subgroups, F5&6 and F7 showed a higher prevalence of BEVs from *Bacteroidota* and *Proteobacteria*, as well as *Actinobacteria*, in contrast to F8&9. On the other hand, F8&9 were characterized by a predominant presence of BEVs from *Firmicutes*.

Figure 8f-g utilize Linear Discriminant Analysis Effect Size (LEfSe) analysis to identify differences between F5&6, F7, F8&9, and between Stool, F8&9, and PF groups. We used a threshold of LDA scores (log10) > 4 and *P* < 0.05 to display statistically significant differences. Most of the phyla and species shown here were previously discussed in Figure 7(a,b), offering strong evidence of their significance. Additionally, this section provides information about taxonomic differences at various levels, such as class, order, family, and more, for reference.

## Discussion

Recent research has suggested that BEVs, which are naturally secreted by the most of bacterial species, may impact both the maintenance of health and the development of diseases, making them significant intermediaries through which the microbiome can influence the host.^29,30^ Despite ample knowledge about the bacterial composition of the gut microbiome, there is still a considerable amount of research needed to fully comprehend the development, attributes, and functions of BEVs in fecal specimens.^20^

This work established three cultured models for two reasons: Firstly, to assess the efficiency of purification by isolating G- BEVs, G+ BEVs, and EEVs from a simplified culture medium. Secondly, to evaluate the effects on functional assays separately for G+ BEVs and G- BEVs within the cultured models, without mutual interference. *E. coli* was chosen as the G- bacteria model because it is a common G- bacterium found in the human colon, and many studies on G- BEVs use it as a model organism.^22,31^ *B. coagulans*, although not a native human gut bacterium, can be consumed through various foods and supplements and has functional properties in the human gut, such as lactic acid production. Additionally, it is easy to culture, making it a suitable representative of G+ bacteria in this study. The Colo205 cell line, derived from human colon cancer, was chosen for the EEVs group. Colo205 cells exhibit gene and protein expression patterns similar to normal colon epithelial cells, making them a valuable model for studying gut biology.^32^ It’s important to note that the human gut microbiota comprises a wide array of both G- and G+ bacteria. Selecting single cultured model can encompass all these types is impossible. Therefore, these cultured models represent subsets of G- BEVs and G+ BEVs. When discussing the effectiveness of distinguishing stool EEVs and BEVs, these three cultured models provided their characteristics such as markers, functional features, etc., in the G- BEVs, G+ BEVs, and EEVs groups as complementary tools.

Some studies have investigated the most appropriate methods for purifying stool-derived BEVs according to the total recovery, reproducibility, purity, and RNA composition of the products; for example, one article mentions five EV separation methods (UC, precipitation [EQ-O, EQ-TC], size exclusion chromatography [SEC], and ultrafiltration [UF]).^33^ However, these methods are not capable of separating BEVs from EEVs. Tulkens et al.^14^ developed a density gradient centrifugation-based purification method for purifying stBEVs. Their data demonstrated that stEEVs typically float at a density of 1.083–1.111 g/mL, while stBEVs have a slightly higher density and are found at 1.133–1.201 g/mL.^14^ Notably, these density ranges correspond to our findings for F5&6 and F8&9. In our results, (G-)*Ec*BEVs were primarily enriched in fractions F8&9, while EEVs showed a higher concentration in fractions F5&6, aligning with previous literature findings. However, the majority of (G+)*Bc*BEVs were found in F5&6, which has not been discussed in the previous study. Based on our TEM data, we observed heterogeneity in size for both EEVs and BEVs. NTA data showed that cultured BEVs (both (G-)*Ec*BEVs and (G+)*Bc*BEVs) were smaller than cultured EEVs, supporting previous findings.^34^ The above results indirectly suggest a higher presence of EEVs in stEVs from F5&6 and a higher presence of G- BEVs in F8&9, but further characterization data are still required for validation.

Secondly, the protein characteristics of these purified EVs were interrogated. Toward this end, well-known exosomal markers, i.e., tetraspanins (CD9), syntenin-1,the ESCRT protein TSG101, the ESCRT-associated protein ALIX, and flotillin, were used.^35^ However, Figure 3a reveals distinct signals for Alix, TSG101, and flotillin in both bacterial lysate and BEVs. It should be noted that bacteria naturally do not express these proteins, which are typically exclusive to eukaryotic systems. This suggests that certain proteins in bacterial lysate and BEVs may nonspecifically bind to these antibodies. Consequently, when assessing the effectiveness of distinguishing stEVs from EEVs and BEVs, the results obtained with these three antibodies are initially disregarded.

When attempting to separate flagella from stEVs in the F8&9 groups of stEVs, we initially employed size exclusion chromatography (SEC). However, the results were not as expected (Figure S2). Based on the WB results, most of the BEVs and Flagellin remain within fractions F3 to F6, without distinct separation. TEM results indicate that EV particle sizes decrease gradually as fraction numbers increase. Nonetheless, Flagella are still present in each fraction, albeit decreasing in size with higher fraction numbers. The coexistence of fragmented flagella with EVs poses challenges for effective separation. Hence, we opted for the IP method to separate Flagella from BEVs.

The LAL test was utilized to quantify the total LPS content and pinpoint the presence of G- BEVs. LPS and (G-)*Ec*BEV were employed as positive control groups to validate that the LAL signal originates from LPS. Furthermore, the results indicated that the most of G- stBEVs were located in F8&9. This finding corroborates the WB results and indicates the presence of LPS in F8&9, with the caveat that the LPS antibody used in WB was unable to detect it.

On the other hand, studies have shown that G+ bacteria, including their BEVs, express LTA and can activate immune cells via TLR2.^14,23,36^ The TLR2 receptor assay employs InvivoGen HEK-Blue™ hTLR2 cells, which are specifically designed for monitoring the activation of NF-kB in response to the stimulation of human TLR2 (hTLR2). When comparing (G+)*Bc*BEVs and (G-)*Ec*BEVs at the same concentration, it’s noteworthy that (G+)*Bc*BEVs exhibit a slightly higher signal. Therefore, it is inferred that G+ BEVs remain the primary influencing factor in this assay.

PBMCs are blood cells that encompass lymphocytes, monocytes, and macrophages. Many of these cells express TLR4, TLR2, and TLR5, which LPS, LTA, or flagella can stimulate to produce proinflammatory cytokines. Therefore, they are suitable for experiments investigating BEVs’ presence.^22^ This study utilized this characteristic to assess whether these fractions of stBEVs can activate human PBMCs. The results showed that all three proinflammatory cytokines (IL-6, TNF-a, and IL-1a) presented the highest values in F8&9. However, when comparing LPS and (G-)*Ec*BEV to F8&9 FT individually, it becomes evident that the group with a higher expression of LPS significantly contributes to this assay (Figure 4g-i, right). Therefore, it is deduced here that G-BEVs containing LPS contribute significantly to the stimulation of PBMCs, while Flagellin and G+ BEVs, although stimulating, do not exhibit the same level of intensity. In summary, stBEVs have a complex role in host cell immune responses. Based on the results of functional studies and precious characterization data, it can be inferred that the most of G+ BEVs and G- BEVs in stBEVs are enriched in the F8&9 fractions.

Subsequently, we explored the optimization of DNA extraction methods for stBEVs and the selection of PCR amplification sites for 16S rRNA genes from stBEV DNA. This study selected two commonly used EV DNA extraction kits (the DNeasy kit, which uses beads to physically disrupt the membrane, and the XCF kit, which uses a lysis reagent to disrupt the membrane). It combined these methods with the Boiling method in an attempt to find the most suitable BEV DNA extraction approach for 16S rRNA sequencing. After PCR amplification, DNA quality control results showed that using the XCF kit directly to extract stBEVs DNA was identified as the most effective method.

PCR primers specific to parts of the 16S rRNA gene containing constant and variable sequences have been used for many years to identify and classify microbiomes. This approach is considered the gold standard for phylogenetic studies.^37^ While selecting the appropriate sequencing method is crucial for precise taxonomic classification, it is equally important to determine the presence of the 16S rRNA gene fragments in stBEVs and assess if specific PCR primers can effectively amplify them. This experiment showed that DNA from stBEVs can be effectively amplified using PCR targeting the V3-V4 or FL region of the 16S rRNA gene. Due to the limited literature discussing the selection of specific 16S rRNA gene amplification regions for stBEVs samples, this study provides empirical validation on this matter.

The metagenomic analysis data of stBEV DNA using the 16S rRNA V3-V4 region and the FL region were compared. Our findings revealed that the FL region group showed a higher abundance of microbial composition at the genus level. Previous studies reported that V3-V4 sequencing of the human gut microbiota resulted in 18.97% of unclassified bacteria at the genus level. In contrast, FL regions sequencing showed only 1.10% of unclassified bacteria. At the species level, V3-V4 sequencing led to a higher proportion of unclassified bacteria at 55.17%, while FL regions exhibited approximately 7.47%. Hence, FL region sequencing is recommended for species-level classification.^38,39^

The differences in microbiota between stool samples and different stBEVs fractions were discussed. Previous studies have reported significant differences in the microbiota composition between gut bacteria and stBEVs, but these studies did not specifically purify stBEVs, which may have led to the sequencing of residual bacterial DNA^40–42^. In the current study, BEVs were purified using a density-based method to ensure the analyzed bacterial DNA was derived from stBEVs. Although the experimental results still showed a significant difference in the microbial composition between gut bacteria and stBEVs, they can serve as a basis for speculating which bacteria are more likely to produce BEVs in the body. At the phylum level, the main microbial components of both the stool samples and stBEVs were *Firmicutes*, *Bacteroidetes*, and *Actinobacteria*, similar to what has been reported in the literature^43^. However, there were significant differences between the two groups at the species level. Currently, there is limited literature on microbiota analysis of stBEVs using FL regions of genes. The variations in purification methods and sequencing approaches may contribute to inconsistent results. Therefore, further data is needed to explore the relationship between gut microbiota and stBEV microbiota. Interestingly, both stBEVs from F8&9 and PF exhibited a similar microbial composition, supporting the effectiveness of our purification method in concentrating the majority of stBEVs in F8&9.

On the other hand, considering that stBEVs possess personalized microbiota composition akin to the gut microbiome (Figure S4), along with their communicative nature and ability to indicate their bacterial origin, these characteristics emphasize the potential of stBEVs as valuable targets for disease categorization and clinical diagnostics. Anticipated future developments are expected to expand the applications of stBEVs even further.

This study has limitations. The gut microbiota is a complex community of trillions of bacteria, archaea, fungi, and viruses. However, in this study, we focused only on three types of EVs: EEVs, G+ BEVs, and G- BEVs. These types may not fully represent the entirety of microbial EVs. It is worth exploring whether archaea, fungi, and viruses can also produce EVs and whether our purification method unintentionally enriches them or related substances. Further investigations are required to gain more insights into these aspects. On the other hand, investigations into gut microbiota are still limited to healthy donors. More diverse samples are needed to better understand the composition of various BEV fractions and their relationship with stool bacteria.

In summary, we optimized a purification method to enrich stBEVs from human stool and validated its efficacy using cultured models representing EEVs, G+ BEVs, and G- BEVs as representative EVs. We employed various techniques to investigate the characteristics of stBEVs, including their particle number, morphology, specific antigens, and functional properties. In addition, we optimized the DNA purification method for stBEVs and established an experimental workflow for microbiota composition analysis. Finally, we established a connection between the gut microbiome composition and stBEVs, with the invention of developing novel fluid-based biomarkers for clinical applications.

## Materials and methods

### Collection of human specimens

Stool samples were obtained with informed consent from healthy individuals and the approval of the Research Ethics Committee Office of the National Taiwan University Hospital under project number 202006060RINB. Three aliquots of stool samples were collected at the same time point and stored in a sterile tube. The samples were transported to the laboratory and stored at − 80°C until isolation, limiting the number of freeze‒thaw cycles to a maximum of one and avoiding under room temperature for more than four hours. Whole blood was collected from donors using a 21 G needle into EDTA-coated vacutainers (BD Biosciences, San Jose, CA). Within 1 hour of collection, the subsequent purification steps for PBMCs were performed as described below.

### Generation of stool pool

Five healthy donors (3 males and 2 females, aged 24–32, with a regular diet) were included for initial protocol optimization. The pooled samples were used for all experiments (Figures 2–5). Each sample was thawed at RT and aliquoted to 5 grams per tube. After the purification method described below, each sample became a crude extract. The crude extracts from five donors were mixed, aliquoted into cryovials, and stored at − 80°C until the next purification step. For the 16S rDNA sequencing analysis, DNA was extracted from each sample. The DNA from five donors was mixed, aliquoted, and stored at −80°C for downstream analyses.

### Cell and Bacterial Culture Medium (CCM & BCM)

#### CCM from the Colo205 cell line

The Colo205 cell line was purchased from the American Type Culture Collection (ATCC, Manassas, VA, USA). Colo205 cells were maintained in RPMI-1640 medium (HyClone SH30027.02) containing 10% FBS and 1% penicillin‒streptomycin (Gibco, CA, USA). For EV isolation, cells were grown in medium containing 10% exosome-depleted FBS (Gibco A27208–01) for 48 h (to ensure that cells reached confluence of ∼70–80%). Thereafter, the CCM was collected and centrifuged at 300 × g for 10 min at RT and then at 1,500 × g for 10 min at 4°C. The subsequent isolation steps for EVs were performed as described below.

#### BCM from the *E.* *coli* DH5α strain and *B.* *coagulans*

The preculture of *E. coli* strain DH5α (native) and *B. coagulans* was carried out by inoculating a single bacterial colony into 5 mL of LB medium (10 g/L tryptone, 5 g/L yeast extract, and 10 g/L NaCl at pH 7). After incubation for 8 h (for DH5α) or 16 h (for *B. coagulans*) at 37°C and 150 RPM, the preculture was inoculated (with 1/100 dilution) into 500 mL of LB medium in a 2-L flask. The culture was collected after several hours (12 h for DH5α and 24 h for *B. coagulans*) of incubation at 37°C and 150 RPM and centrifuged at 600 × g for 15 min at RT and then at 1,500 × g for 10 min at 4°C. The subsequent isolation steps for EVs were performed as described below.

### Isolation of EVs from CCM, BCM and human feces using DG and UC methods

The EV purification method was described previously^14^ with minor modifications. An overview of sample preparation is provided in Figure 1. Briefly, five grams of stool sample was dissolved in 50 mL of prewarmed (37°C) filtered phosphate-buffered saline (PBS) in a 50 mL sterile centrifugal tube. To suspend the sample gently, constant 360° rotation was applied in a slight vibration mode on the sample mixer (HulaMixer, Invitrogen) for 30 min at 37°C. After these steps, CCM and BCM underwent the same preparation protocol described below. The sample was centrifuged at 8,000 × g for 15 min at 4°C to remove cell debris, fibers, bacteria, and undigested food. The supernatant was collected in a new 50 mL sterile centrifugal tube and then centrifuged at 12,000 × g for 15 min at 4°C to remove contaminants, then filtered through a 0.22-μm Nalgene Rapid-Flow filter (Thermo Fisher Scientific, Waltham, MA, USA) done on the ice. The sample was concentrated by using 100-kDa Amicon Ultra15 centrifugal filters (Merck) at 5,000 × g for 8 min several times. Finally, samples were concentrated to a volume of at least 1350 μl, distributed in aliquots of 450 µl per cryovial, and stored at −80°C until the test. The crude extract was mixed with iodixanol (OptiPrep, Sigma #1556) to obtain a final proportion of 50% (w/v) iodixanol. The 2.68 mL mixture was transferred to a tube bottom, followed by loading with a 2.68 mL 40%, 2.68 mL 20%, and 2.35 mL 10% iodixanol gradient; these dilutions were prepared by diluting a stock solution (60% w/v) of iodixanol with a buffer solution (0.25 M sucrose/1 mM EDTA/10 mM Tris, pH 7.4), and the top was loaded with 0.67 mL filtered PBS. The tubes were then centrifuged at 100,000 × g for 18 h at 4°C in a P40ST swinging bucket rotor (Hitachi CP80 WX). Thirteen fractions of 670 µl each were collected, starting with the top fraction. All fractions were diluted and washed with filtered PBS and centrifuged at 100,000 × g for 3 h at 4°C in the MLA-50 rotor (BECKMAN). Finally, each fraction was resuspended in 200 µl of PBS.

### Size exclusion chromatography (SEC) for separating flagella and BEVs in F8&9 of stEV samples

Ten milliliters of sepharose beads CL-2B (GE Healthcare) were allowed to settle in a PD-10 column (GE Healthcare). They were washed five times with endotoxin-free PBS, using a volume equal to five times the bead’s volume. After washing, the beads were packed. F8&9 from the samples, previously subjected to DG, were collected. Approximately 2 mL of this fraction was loaded into the column. The liquid that dripped down was collected in 1 mL eppendorf tubes, totaling 15 tubes. Each tube underwent UC to enrich and remove the density buffer. Finally, the purification results were analyzed using WB and TEM.

### Immunoprecipitation for separating flagella and BEVs in F8&9 of stEVs, (G-)*Ec*BEVs, and (G+)*Bc*BEVs samples

The flagellin antibody was coupled using the Dynabeads™ Protein G Immunoprecipitation Kit (Invitrogen) following the provided instructions. In brief, 50 μL of Dynabeads™ magnetic beads were resuspended, mixed with 10 μg of the anti-flagellin antibody (Abcam) in 200 μL of Ab Binding and Washing Buffer. The mixture was rotated at room temperature using a HulaMixer™ Sample Mixer for 30 minutes. Subsequently, it was washed twice with the same buffer using gentle pipetting. Additionally, the crosslinking reagent DSS (Invitrogen) was used as per the manual to prevent co-elution of the conjugated antibody. Antibody-coupled magnetic beads were added to the 200 μL sample and incubated at 4°C for 2 hours on a rotor. The mixture was then placed on a magnet for 1 minute, resulting in the supernatant known as the F8&9 Flow-through (FT). The beads underwent three washes, each with 200 μL of Washing Buffer. Subsequently, 20 μL of Elution Buffer was added, and the magnetic bead-Ab-Ag complex was gently pipetted to resuspend it, and this sample is referred to as F8&9 Pull Down (PD). Finally, both FT and PD samples were processed using a 10 kDa ultrafiltration centrifugation to switch to an endotoxin-free PBS buffer, preparing them for the subsequent functional assay.

### Nanoparticle Tracking Analysis (NTA)

NTA was used to determine the EV size distribution and concentration with a NanoSight NS300 instrument (Malvern) equipped with a 488 nm blue laser and an sCMOS camera. NanoSight software (NTA 3.4 Build 3.4.003) was used for recording and analysis. Briefly, each EV sample was diluted with PBS according to the detection range (20–100 particles/frame). For each sample, six 60-second videos were captured with the following settings: syringe flow rate 100, camera level 16, temperature 25°C, and viscosity 0.9 cP (water). For analysis, the following settings were used: screen gain 10 and detection threshold 4. The focus was automatically adjusted daily using standard beads.

### Transmission Electron Microscopy (TEM)

The EV pellet was resuspended in PBS before being assessed by TEM. A 10 µl sample was absorbed on a carbon-coated 200 mesh copper grid for 5 min, followed by another round of fixation with 2.5% (w/v) glutaraldehyde for 5 min. The grids were washed with phosphate buffer six times for 1 min each and with distilled water twice for 1 min each. After negative staining with 2% (w/v) uranyl acetate for 1 min, the remaining dye was immediately removed with a filter paper. Thereafter, the grids were observed with a Hitachi H-7650 transmission electron microscope operated at 120 kV.

### Western blot analysis

Protein concentrations were determined using the Pierce BCA protein assay kit (Thermo Scientific, Waltham, MA, USA). Two sample loading strategies were employed: 10 μg protein was loaded for fractions with sufficient protein (usually F5&6, F7, and F8&9), while 30 μl of the sample was loaded for other fractions with less than 10 µg per 30 μl. All samples were prepared in 4× Laemmli sample buffer (Bio-Rad, Benicia, CA, USA) containing β-mercaptoethanol and heated at 95°C for 15 min. Samples were separated on 4–15% Mini-PROTEAN TGX precast Gels (Bio-Rad) on a Mini PROTEAN Tetra Cell system (Bio-Rad) and transferred to an Immobilon-P PVDF membrane (Millipore) following the manufacturer’s guidelines. The membrane was then blocked with 5% BSA for 1.5 h at RT and incubated overnight at 4°C with the primary antibodies. After incubation with primary antibodies, the membranes were washed with 0.05% Tween in phosphate-buffered saline (PBST) six times for 5 min each. Secondary antibody staining was conducted at RT for 1 h. After washing with PBST, the membranes were detected using Western Lightning Ultra substrate (PerkinElmer). Images were taken by using a UVP ChemStudio PLUS Touch system (Analytic Jena). All the antibodies used in this study are listed in Supplementary Table .

### Biochemical endotoxin detection by Limulus Amebocyte Lysate (LAL) test

For the stEVs group, each sample was diluted to 10^6^ particles in a 50 μl volume per well. The F8&9 PD and F8&9 FT samples were prepared using the same protein concentration (diluted to approximately 0.000625 µg/mL). LPS was used at a standard concentration of approximately 1.5 EU/mL, and (G+)*Bc*BEVs and (G-)*Ec*BEVs were employed at the same particle concentration as stEVs group. Testing was performed with the Pierce Chromogenic Endotoxin Quant Kit (Thermo Scientific) by following the manufacturer’s guidelines. In brief, 50 μl of the sample, endotoxin standard dilutions, and endotoxin-free water were added to the prewarmed plate. Furthermore, 50 μl of Amebocyte Lysate Reagent was added, followed by incubation for 12 min at 37°C. Thereafter, 100 μl of prewarmed chromogenic substrate solution was added, followed by incubation for 6 min at 37°C. Finally, 25% acetic acid was added to stop the reaction, and the optical density (OD) at 405 nm was measured using SpectraMax iD3 Multi-Mode Microplate Readers (Molecular Device, Silicon Valley, CA, USA).

### Peripheral Blood Mononuclear Cell (PBMC) stimulation test

#### Isolation of PBMCs from human peripheral blood

Peripheral blood from healthy donors was collected in an EDTA-coated tube (BD Biosciences, San Jose, CA, USA.). The whole blood was diluted with 2 times the volume of PBS/EDTA (with 2 mM disodium EDTA). Fifteen milliliters of Ficoll-Paque PREMIUM sterile solution with a density of 1.077 (Cytiva) was loaded in a 50 mL centrifuge tube, and 30 mL of the diluted blood was carefully layered on Ficoll-Paque solution. The tube was centrifuged at 400 × g for 40 min at RT. The mononuclear cell fractions at the interface between plasma and Ficoll-Paque were collected and transferred to a new tube. The pellet was resuspended in 40 mL of PBS/EDTA and centrifuged at 300 × g for 10 min at RT. The supernatant was discarded, and PBMCs were resuspended in 5 mL of PBS.

#### PBMC stimulation test

PBMCs were diluted to 1.33 × 10^7^ cells/ml with prewarmed (37°C) RPMI-1640 medium (HyClone SH30027.02) supplemented with 5% exosome-depleted FBS (Gibco A27208–01). Then, 150 μl of PBMC solution was added to a well of a 96-well plate, and PBMCs were allowed to adhere for 2 h (37°C, 5% CO2). The cell medium was carefully removed, and prewarmed serum-free RPMI medium was replaced twice; For stEVs, (G+)*Bc*BEVs, and (G-)*Ec*BEVs, BEVs were diluted to a concentration of 2 × 10^7^ BEVs/mL in serum-free RPMI medium and added to the wells. F8&9 PD and F8&9 FT samples were added to wells at the same protein concentration (diluted to approximately 0.0125 µg/mL). LPS was added at a standard concentration of approximately 1.5 EU/mL. The samples were then incubated for a minimum of 24 h.

Thereafter, the medium was collected and centrifuged at 500 × g for 9 min at 4°C to remove cell debris. The BEVs were removed by ultrafiltration with a 100-kDa centrifugal filter at 3,000 × g for 10 min at 4°C. The flow-through was concentrated with a 3-kDa centrifugal filter. IL-1α, TNF-α, and IL-6 Quantikine ELISA kits (R&D system) were used to measure cytokine contents according to the instructions provided by the manufacturer.

### TLR2 receptor assay

TLR2 activity was measured using HEK-Blue^TM^ hTLR2 cells (InvivoGen) according to the instructions provided by the manufacturer. In brief, HEK-Blue hTLR2 cells were maintained in medium supplemented with 1X HEK-Blue Selection (InvivoGen) to 50–80% confluence. The cells were separated from the growth medium and rinsed with prewarmed PBS twice to remove cell debris. The cells were suspended in HEK-Blue Detection medium (InvivoGen) and diluted to 2.8 × 10^5^ cells/mL. Then, 180 μl of the cell suspension was immediately added to each well of the 96-well plate. For stEVs, (G+)*Bc*BEVs, and (G-)*Ec*BEVs, the samples were prepared by diluting BEVs with prewarmed PBS to a concentration of 1 × 10^8^ BEVs/mL and adding them to the wells. F8&9 PD and F8&9 FT samples were added to wells using the same protein concentration (diluted to approximately 0.0625 µg/mL). LPS was added at a standard concentration of approximately 1.5 EU/mL. Twenty microliters of a diluted sample were added, followed by gentle mixing and incubation at 37°C in CO2 for 18 h. The assay results were obtained by measuring the optical density (OD) at 620 nm by using SpectraMax iD3 Multi-Mode Microplate Readers.

### Strategy for extracting stBEVs DNA and amplifying the 16S rRNA gene

Figure 5a depicts this whole procedure. In the DNA purification strategy for stBEVs, we considered the following five methods: (1) Boiling: involved boiling purified stool EVs at 100°C for 40 minutes, followed by centrifugation at 13,000 × g for 30 minutes at 4°C and collection of the supernatant. (2) Boiling followed by DNA extraction using the XCF Exosomal DNA isolation kit (Boiling+XCF), (3) Boiling followed by DNA extraction using the DNeasy PowerSoil Pro kit (Boiling+DNeasy), (4) Direct DNA extraction using the XCF kit (XCF) and (5) Direct DNA extraction using the DNeasy kit (DNeasy). At Checkpoint 1, the total DNA yield extracted from the five methods mentioned above was measured using the Qubit 2.0 Fluorometer. The top two methods with the highest DNA yield were selected for further analysis. Next, DNA purified from the two selected DNA extraction methods was subjected to PCR amplification targeting the V3-V4 and FL regions of the 16S rRNA gene. At Checkpoint 2, the total DNA yield from the amplified DNA was quantified using the Nanodrop system. Additionally, the quality of the PCR-amplified DNA was assessed using gel electrophoresis. After passing Checkpoint 2, the selected samples proceeded to the subsequent experiment of 16S rRNA sequencing.

### Extraction of stool and stool bacteria DNA

DNA extraction from stool and stool bacteria was performed using the DNeasy PowerSoil Pro kit according to the manufacturer’s instructions. For stool samples, 250 mg of sample was directly used as per the kit’s instructions. Stool bacteria samples were prepared by centrifuging 250 mg of stool in 50 mL of endotoxin-free PBS at 6000 g for 30 minutes to remove the EV-containing supernatant. This step was repeated twice, and then the pellet was processed for DNA extraction following the kit’s instructions.

### DNase I treatment of DNA from bacterial lysate and stBEVs

The DNase I treatment experiment was conducted using the TURBO DNA-free kit (Invitrogen), following the manufacturer’s instructions. Two groups for DNA from bacterial lysate and two groups for DNA from stBEVs were tested with equal DNA concentrations (approximately 15 ng/µL). Following the routine DNase treatment protocol from the kit’s instructions, samples were then purified using the XCF Exosomal DNA isolation kit. Subsequently, PCR targeting the FL region was performed, followed by electrophoresis testing.

### FL region of 16S rRNA amplification and gene sequencing on the PacBio platform

16S gene-specific primers with barcodes were used to amplify FL 16S genes (V1-V9 regions). Each primer contained a 5’ phosphate-modified buffer sequence (GCATC), a 16-base barcode, and degenerate 16S gene-specific forward or reverse primer sequences (Forward: 5’Phos/GCATC-16-base barcode-AGRGTTYGATYMTGGCTCAG-3’, Reverse: 5’Phos/GCATC-16-base barcode-RGYTACCTTGTTACGACTT-3’, where the degenerate base identities used were *R* = A, G; Y = C, T; and *M* = A, C). PCR was carried out with KAPA HiFi HotStart ReadyMix (Roche) using 2 ng of gDNA, and the products were assessed on a 1% agarose gel. Samples with a bright main band at approximately 1500 bp were purified using AMPure PB Beads and used for subsequent library preparation. The PCR conditions were as follows: 95°C for 3 minutes; 20 ~ 27 cycles of 95°C for 30 seconds, 57°C for 30 seconds, 72°C for 60 seconds; 72°C for 5 minutes; and holding at 4°C. The SMRTbell library was prepared using the amplification of FL 16S gene with barcoded primers for multiplexed SMRTbell library preparation and sequencing procedure (PacBio). The barcoded PCR products were pooled in equal amounts, and 500–1,000 ng of the pooled amplicon sample was subjected to DNA damage repair, followed by end-repair/A-tailing and ligation to introduce universal hairpin adapters to double-stranded DNA fragments. The SMRTbell library was purified with AMPure PB beads to remove adapter dimers and then incubated with sequencing primer v4 and Sequel II Binding Kit 2.1 reagents for primer annealing and polymerase binding. Finally, sequencing was performed in circular consensus sequence (CCS) mode on a PacBio Sequel IIe instrument to generate HiFi reads with a predicted accuracy (Phred scale) = 30.

### V3-V4 region of 16S rRNA amplification and gene sequencing on the Illumina platform

The V3-V4 region of the 16S rRNA gene was sequenced using specific primers (341F: 5’-CCTACGGGNGGCWGCAG-3’, and 806 R: 5’- GACTACHVGGGTATCTAATCC-3’) according to the 16S Metagenomic Sequencing Library Preparation protocol (Illumina). PCR was carried out using 12.5 ng of gDNA with KAPA HiFi HotStart ReadyMix (Roche) under the following conditions: 95°C for 3 minutes; 25 cycles of 95°C for 30 seconds, 55°C for 30 seconds, 72°C for 30 seconds; 72°C for 5 minutes; and holding at 4°C. PCR products with a bright main band at approximately 500 bp were selected and purified with AMPure XP beads for library preparation. The sequencing library was prepared using the 16S Metagenomic Sequencing Library Preparation procedure (Illumina). Secondary PCR was conducted with the V3-V4 region PCR amplicon and the Nextera XT Index Kit, containing dual indices and Illumina sequencing adapters. Indexed PCR product quality was evaluated using a Qubit 4.0 Fluorometer and a Qsep100TM system. The indexed PCR products were mixed in equal amounts to generate the sequencing library. Finally, paired 300-bp reads were generated using the Illumina MiSeq platform.

### Taxonomic assignment

#### For the PacBio platform

PacBio’s official workflow with a minimum of three passes and a predicted accuracy of 0.9 was used to generate circular consensus sequences (CCSs), which were demultiplexed and further processed using DADA2.^36^ The DADA2 workflow included quality filtering, dereplication, learning the dataset-specific error model, ASV inference, and chimera removal. The algorithm resolved exact amplicon sequence variants (ASVs) with a single-nucleotide resolution and a near-zero error rate. Taxonomic classification was performed using the feature-classifier^44^ and classify-consensus-blast algorithms in QIIME2,^45^ with information retrieved from the NCBI database. Multiple sequence alignment was conducted using MAFFT^46^ against the NCBI database, and a phylogenetic tree was constructed using the QIIME2 phylogeny FastTree^47,48^ algorithm with a set of representative ASV sequences.

This study examined α-diversity indexes, including species richness indicators such as observed features, Menhinick, and Margalef; as well as species diversity indices such as Shannon entropy, Simpson, Pielou’s evenness, and Faith’s phylogenetic diversity.

In β-diversity analysis, Constrained Principal Coordinate Analysis (CPCoA) were employed. CPCoA enhances Principal Coordinate Analysis (PCoA) by considering the links between factors of interest and species composition. It merges PCoA with Redundancy Analysis (RDA) and incorporates group information to find the planes that best explain inter-group differences in specific conditions. It assesses sample similarity and quantifies how group information contributes to sample variations. The statistical significance (P) was determined through anova.cca with permutation tests. If P is statistically significant, it indicates that group information can explain part of the variation among samples.

#### For the Illumina platform

Amplicon sequencing generated 300 bp paired-end reads, which were demultiplexed based on barcodes. The QIIME2 Cutadapt plugin was used to remove primer and adapter sequences. The QIIME2 DADA2 plugin was used to construct ASVs, which involved quality filtering, dereplication, error model learning, denoising, paired-end read joining, and chimera removal. A maximum of two expected errors per read was set for trimming and filtering. Taxonomic classification was performed using the feature-classifier algorithm in QIIME2 and the Silva database. Multiple sequence alignment was conducted with QIIME2 alignment MAFFT against the Silva database, and a phylogenetic tree was constructed with QIIME2 phylogeny FastTree using representative ASV sequences.

### Statistical analyses

The data were analyzed using either one-way ANOVA or Student’s t test at a significance level of *p* < 0.05. GraphPad Prism 9.0 software was used for analysis, and the results are presented as the mean ± SD for three independent experiments. Welch’s t-test is employed to compare means and determine if two different, randomly sampled, independent datasets have equal average values. To identify species with significant differences between pairs of categories, the Welch’s t-test is used for inter-group analysis. Species with significant differences (p-value <0.05) are identified and illustrated in inter-group species variation bar charts. Furthermore, we employed LEfSe (Linear Discriminant Analysis Effect Size), which uses non-parametric factorial Kruskal-Wallis sum-rank tests to detect species with significant abundance differences. Linear discriminant analysis (LDA) was then applied to estimate the magnitude of the effect of each species’ abundance on the differentiation. This approach helps identify communities or species that significantly influence the division of samples. To display statistically significant differences, we utilized a threshold of LDA scores (log10) > 4 and *P* <0.05.

### Generation of schematic diagrams

All schematic diagrams in this article were created using biorender.com.

## Supplementary Material

Supplemental Material

## Data Availability

Data used to in this work will be made available upon request and is conditional to approval from the Graduate Institute of Biomedical Electronics and Bioinformatics at National Taiwan University.
