# Supplementary material for: Characterization of markers, functional properties, and microbiome composition in human gut-derived bacterial extracellular vesicles
Source: Gut Microbes. 2023 Dec 1;15(2):2288200. doi: 10.1080/19490976.2023.2288200 (PMC10730231; doi:10.1080/19490976.2023.2288200)
Supplement: Supplemental Material [file KGMI_A_2288200_SM2048.docx]

*Supplemental material*

*For*

**Characterization of markers, functional properties, and microbiome composition in human gut-derived bacterial extracellular vesicles**

This document contains supplementary figures and tables related to the main text.

**Contents**

**Figure S1.** Illustration of OptiPrep density gradient centrifugation and ultracentrifugation results for human stool EVs.

**Figure S2.** The size exclusion chromatography (SEC) method is ineffective in separating stEVs from Flagella.

**Figure S3.** From five healthy individuals, the microbiome in stool, stool bacteria, and various stBEV fractions were analyzed by metagenomic analysis.

**Figure S4.** stBEVs derived from different individuals can show significantly differences in their characteristics and origin, even under identical experimental conditions.

**Table S1.** Information of used antibodies


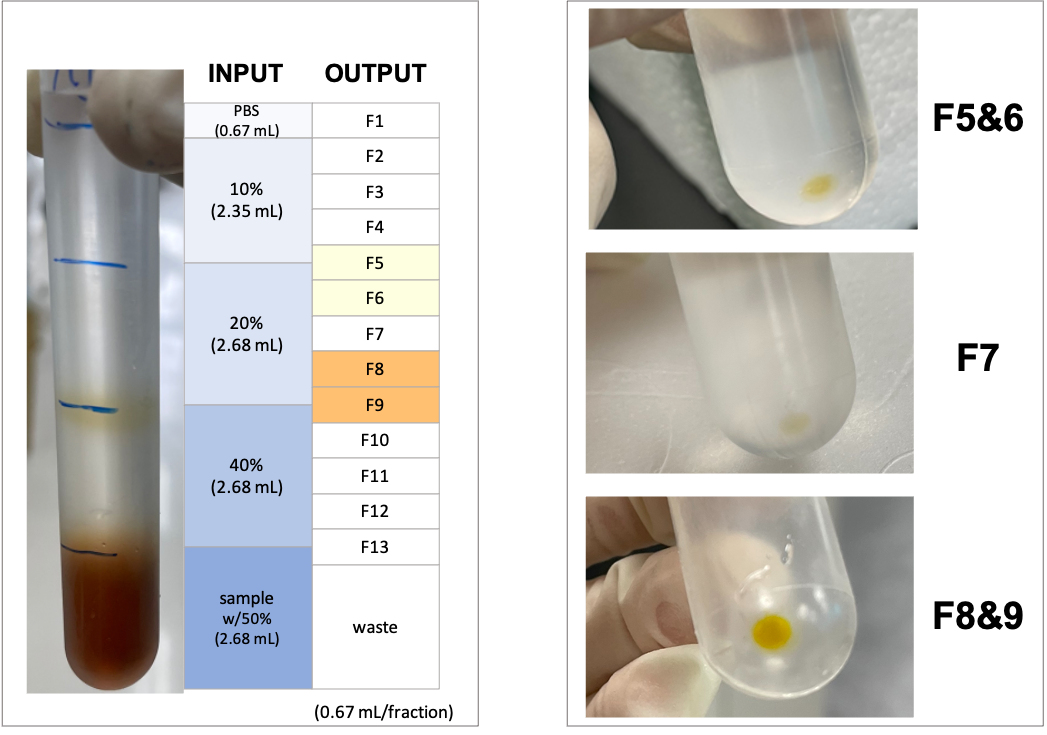


**a**

**b**

**FIGURE S1** Illustration of OptiPrep density gradient centrifugation and ultracentrifugation results for human stool EVs. (a) Diagram of the experimental design for OptiPrep density gradient fractionation, including sample injection and collection, and (b) representative image of the pellets obtained after ultracentrifugation of fractions F5&6, F7, and F8&9. No pellets were observed in the other fractions.


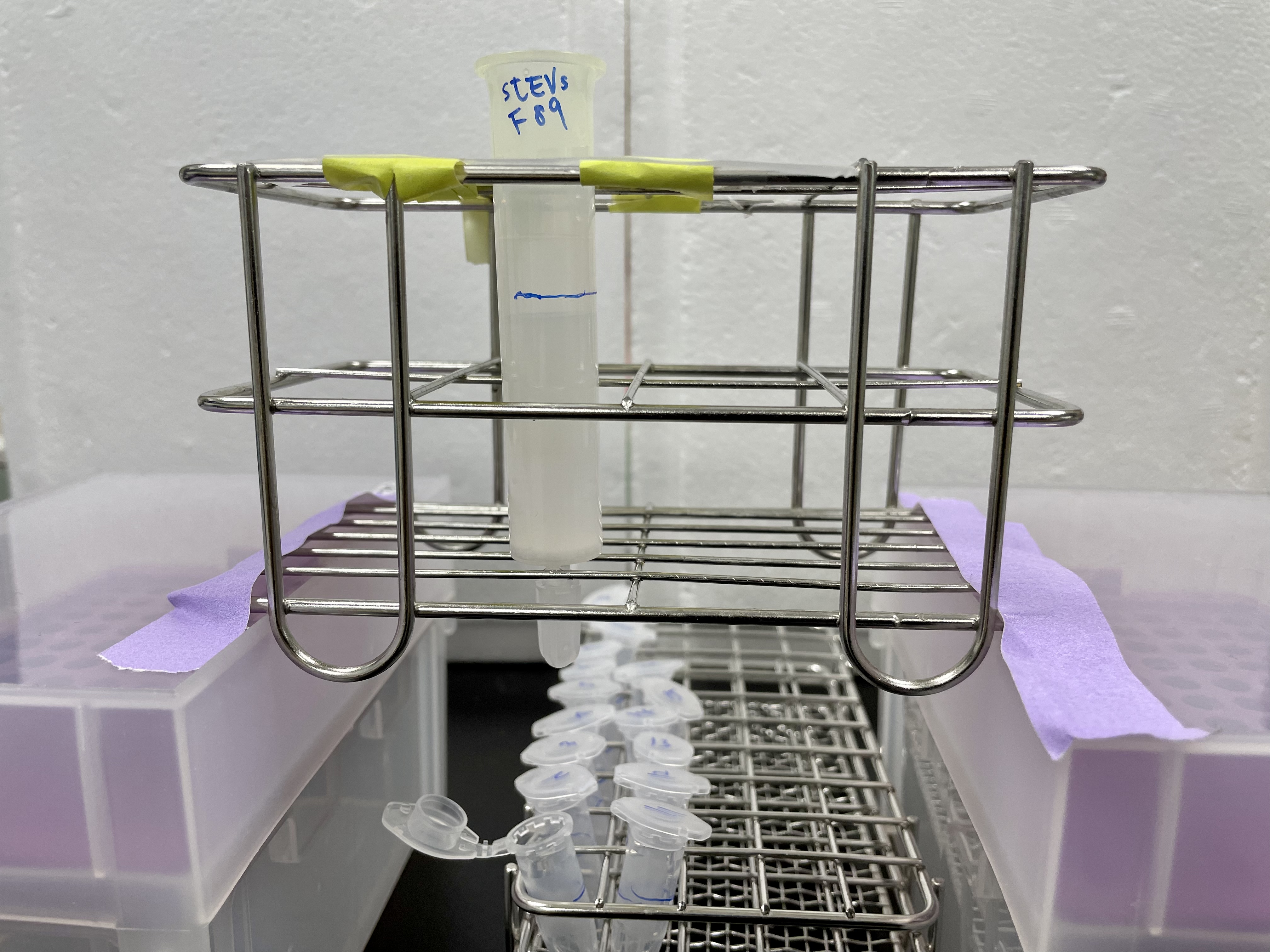

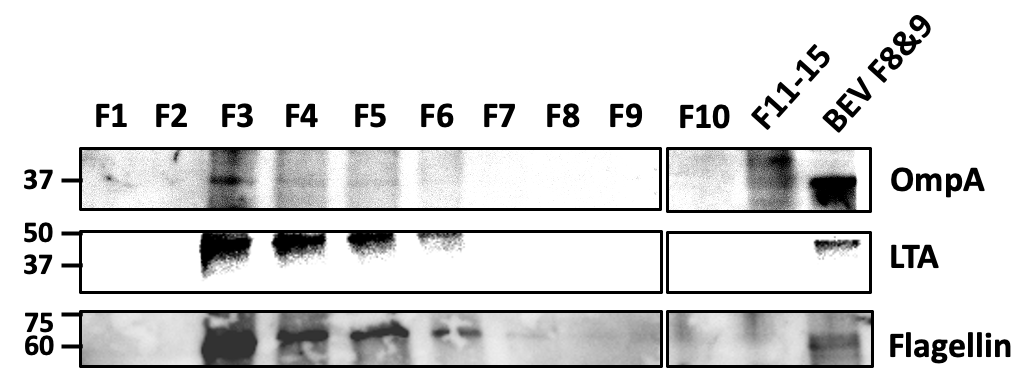

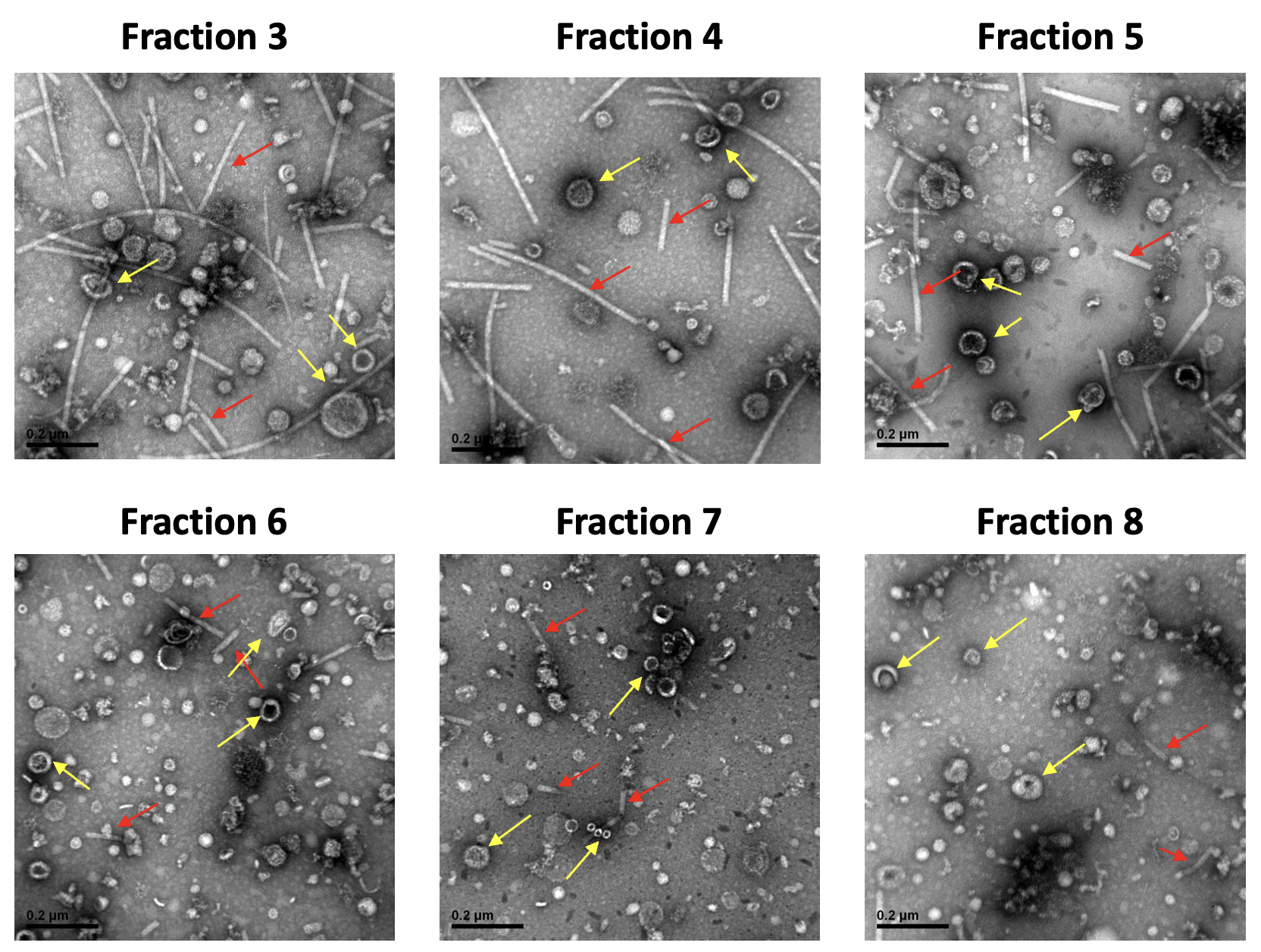


**a**

**b**

**c**

**FIGURE S2** The size exclusion chromatography (SEC) method is ineffective in separating stEVs from Flagella. (a) This figure demonstrates the use of 10 mL washed CL-2B Sepharose beads and packed columns, with 1 mL collected in tubes. The experiment utilized stBEV F8&9 fractions directly extracted from a density gradient. Subsequently, each SEC-separated fraction underwent a 3 h ultracentrifugation to remove the Iodixanol. (b) Fractions 3 to 5 were loaded at 10 µg/well, and fractions with lower concentrations were loaded at 30 µL/well. Testing covered F1-F10, F11~F15, and untreated stBEV F8&9. OmpA served as a marker for G-BEVs, LTA for G+ BEVs, and Flagellin for Flagella. (c) Representative TEM images show results from fractions 3 to 8, with a scale bar of 200 nm. Yellow arrows highlight EVs, while red arrows indicate flagella or pili fragments.


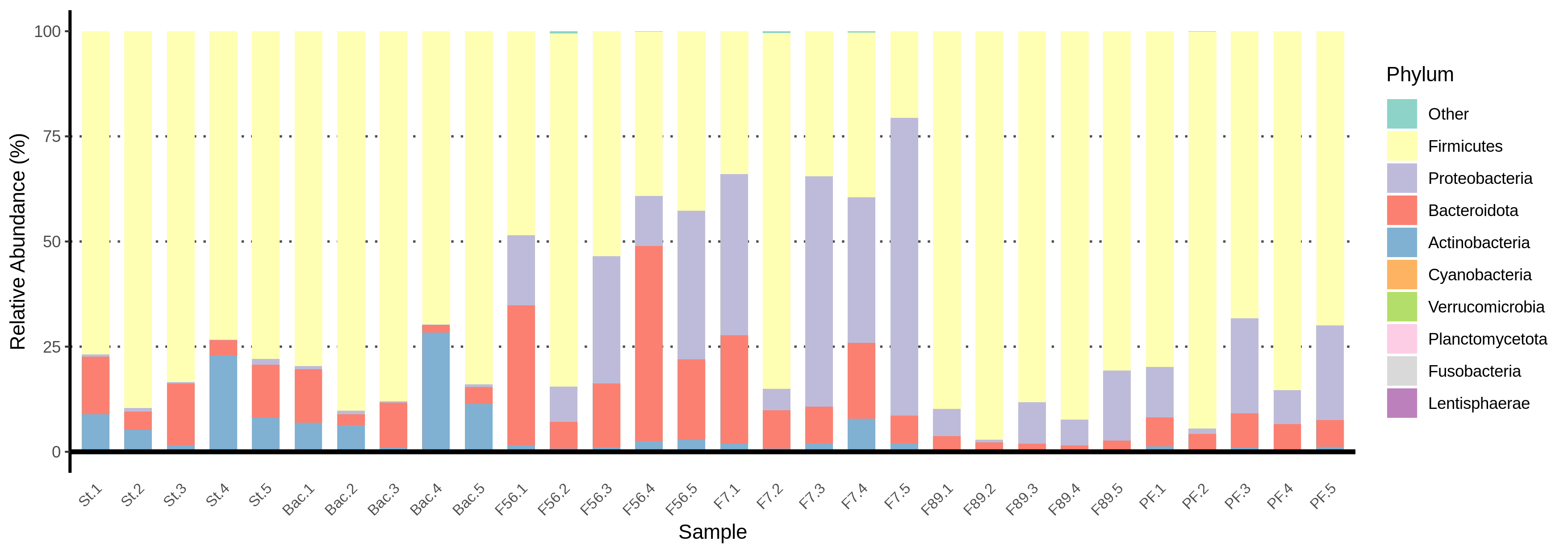

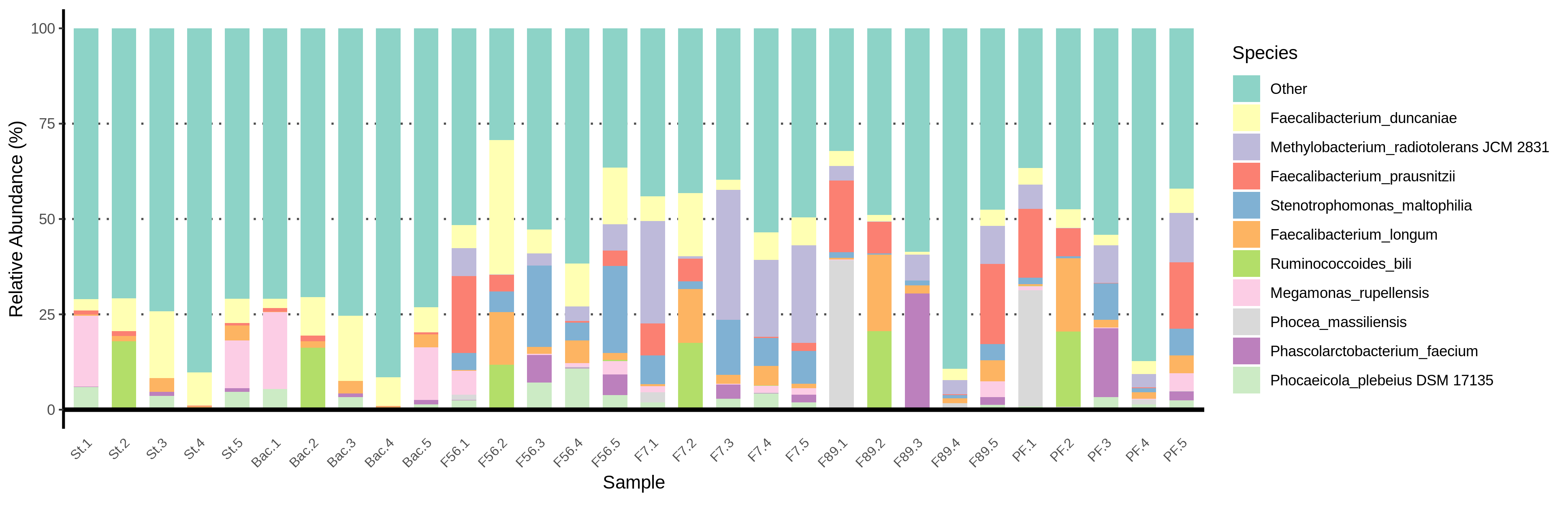


**a**

**b**

**FIGURE S3** From five healthy individuals, the microbiome in stool, stool bacteria, and various stBEV fractions were analyzed by metagenomic analysis. In five healthy individuals, this figure displays the comparative relative abundance (% of total 16S rDNA gene sequences) of gut microbes at the phylum level (a) and species level (b) in stool (St), stool bacterial lysate (Bac), and stBEV fractions: F5&6 (F56), F7, F8&9 (F89), and pooled-fractions F5-F9 (PF).


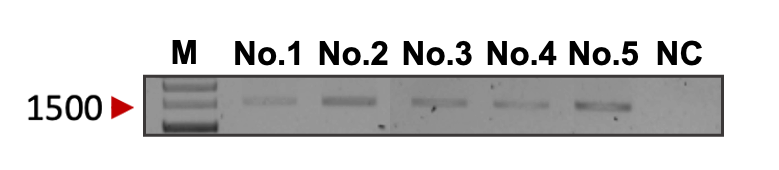

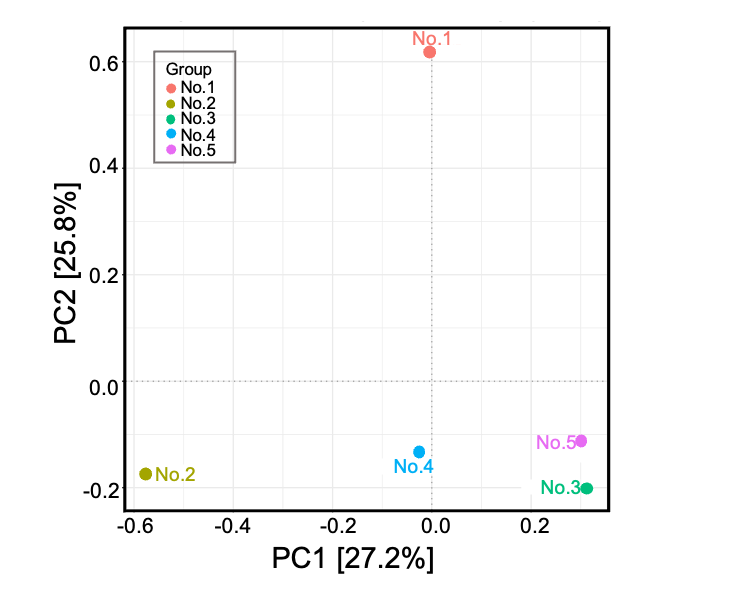

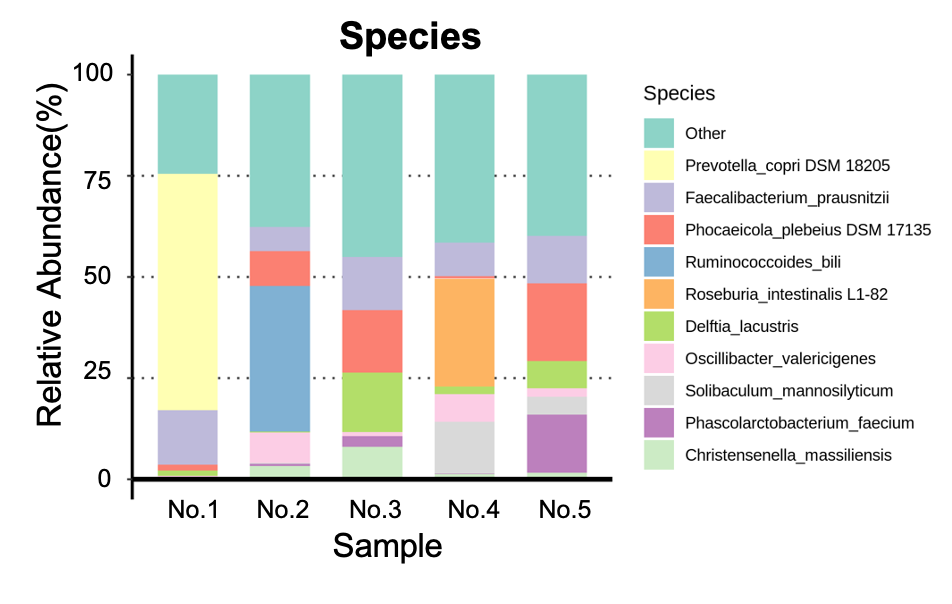


**a**

**b**

**c**

**d**

**e**

**f**

**FIGURE S4** stBEVs derived from different individuals can show significantly differences in their characteristics and origin, even under identical experimental conditions. (a, b) Equal amounts of stBEVs were extracted from five healthy individuals (no. 1- no. 5), and their DNA was amplified by targeting the FL region of 16S rRNA. The DNA quality was observed by DNA electrophoresis and DNA was quantified using a NanoDrop system. (c) The figure shows the α-diversity indices for the five sequenced samples. (d) The β diversity indices describe the interrelationships between pairwise samples' microbiota. (e) The figure illustrates the β diversity based on the Bray-Curtis dissimilarity for the five sequenced samples using a PCA plot. (f) The figure displays the top 10 bacterial species and their relative abundance (%) at the species level for the five sequenced samples.

**Supplementary Table 1. Information of used antibodies**
